# Positive Reciprocal Feedback of *lncRNA ZEB1-AS1* and *HIF-1*α Contributes to Hypoxia-Promoted Tumorigenesis and Metastasis of Pancreatic Cancer

**DOI:** 10.3389/fonc.2021.761979

**Published:** 2021-11-22

**Authors:** Yan Jin, Zhengming Zhang, Qiao Yu, Zhu Zeng, Hong Song, Xiaoxu Huang, Qi Kong, Hao Hu, Yabin Xia

**Affiliations:** ^1^ Department of Gastrointestinal Surgery, The First Affiliated Yijishan Hospital of Wannan Medical College, Wuhu, China; ^2^ Department of Gastroenterology, Union Hospital, Tongji Medical College, Huazhong University of Science and Technology, Wuhan, China; ^3^ Department of Emergency Surgery, Union Hospital, Tongji Medical College, Huazhong University of Science and Technology, Wuhan, China; ^4^ Department of Pathology, The First Affiliated Yijishan Hospital of Wannan Medical College, Wuhu, China

**Keywords:** pancreatic cancer, *lncRNA-ZEB1-AS1*, *Zeb1*, *HIF-1α*, metastasis, hypoxia

## Abstract

**Background:**

Many studies have reported the roles of the extracellular hypoxia microenvironment in the tumorigenesis and metastasis of multiple cancers. However, long noncoding RNAs (*lncRNA*s) that induce cancer oncogenicity and metastasis of pancreatic cancer (PC) under hypoxia conditions remain unclear.

**Methods:**

In PC cells, the expression levels of *lncRNA*s in different conditions (normoxia or hypoxia) were compared through RNA sequencing (RNA-seq). The effects of the zinc finger E-box-binding homeobox 1 (*ZEB1-AS1*) antisense *lncRNA* on PC cells cultured in normoxia/hypoxia medium were measured through gain and loss-of-function experiments. Fluorescence *in situ* hybridization and luciferase reporter assays in addition to *in vivo* studies were utilized to explore the adaptive mechanisms of *ZEB1-AS1* in the hypoxia-promoted proliferation, migration, and invasion ability of PC cells. Moreover, the level of *ZEB1-AS1* and its associated targets or pathways were investigated in both PC and pancreatic normal tissues.

**Results:**

RNA-seq revealed that *ZEB1-AS1* was significantly upregulated in PC cells under hypoxia conditions. The *ZEB1-AS1* expression level was closely associated with poor prognosis of PC patients. Knockdown of *ZEB1-AS1* suppressed the proliferation, migration, and invasion of PC cells *in vitro* as well as PC xenograft tumor growth *in vivo*. In PC cells, RNAi-mediated reduction of *ZEB1-AS1* inhibited zinc finger E-box-binding homeobox 1 (*ZEB1*), while *ZEB1-AS1* overexpression rescued *ZEB1* expression, indicating that *ZEB1-AS1* promotes *ZEB1* expression. Moreover, hypoxia-inducible factor-1α (*HIF-1*α)induced the expression of *ZEB1-AS1* by binding to the *ZEB1-AS1* promoter, which contains a putative hypoxia response element (HRE). Mechanistically, *ZEB1-AS1* scaffolded the interaction among *HIF-1*α, *ZEB1*, and histone deacetylase 1 (*HDAC1*), leading to deacetylation-mediated stabilization of *HIF-1*α. We further revealed that *ZEB1* induced the deacetylase capacity of *HDAC1* to suppress the acetylation or degradation of *HIF-1*α, improving *HIF-1*α assembly. Thus, hypoxia-induced *ZEB1-AS1* facilitated *ZEB1* transcription and the stability of *HIF-1*α, which promoted the metastasis of PC cells. Clinically, dysregulated *ZEB1* and *HIF-1*α expression was significantly correlated with histological grade, lymphatic metastasis, and distant metastasis in PC patients.

**Conclusions:**

Our results emphasized that the positive reciprocal loop of *HIF-1*α/*ZEB1-AS1*/*ZEB1*/*HDAC1* contributes to hypoxia-promoted oncogenicity and PC metastasis, indicating that it might be a novel therapeutic target for PC.

## Introduction

Pancreatic cancer (PC) is often diagnosed at advanced stages of the disease when the treatment options are limited and consequently lead to the poor overall patient survival rates (1). PC is one of the most lethal types of cancer with increasing incidence and mortality rates worldwide (2, 3). Early invasion and metastasis are the main factors that challenge the treatment of these patients (4). Therefore, the potential molecular mechanism of the oncogenicity and metastasis ability of PC needs to be discovered and elucidated.

Long noncoding RNAs (*lncRNA*s) are noncoding RNAs that are more than 200 nucleotides in length, and they may be pivotal regulators of oncogenesis, indicating their potential in therapeutic strategies for improving the clinical outcomes of PC patients (5, 6). Accumulating evidence has suggested the role of *lncRNA*s in human disease etiopathogenesis, including cell differentiation, cell cycle progression, epigenetic regulation and malignant neoplasms. Takahashi et al. revealed that *lncRNA-HULC*, which is suppressed by *microRNA-133b*, accelerates PC cell invasion, migration and *EMT* (7). Liu et al. showed that accumulation of *lncRNA-CF129* inhibits pancreatic cell oncogenicity *via MKRN1*-induced ubiquitin-dependent *p53* degradation following transcriptional suppression of *FOXC2* (8). Other studies have reported that *lncRNA GLS-AS* and *lncRNA MTSS1-AS* influence the modulation of PC progression (9, 10). Our previous findings demonstrated the crucial role of *lncRNA-NUTF2P3* in inducing *KRAS* levels by participating in *miR-3923* knockdown in PC (11). In addition, we also reported that *lncRNA-MTA2TR* enhances PC by depriving acetylation and aggregation of hypoxia-inducible factor-1α (*HIF-1*α) (12). These studies establish that *lncRNA*s play notable roles in mediating PC initiation and progression. Nevertheless, the roles of *lncRNA*s in PC need further investigation to explore the underlying mechanism and enrich the therapeutic targets for PC patients.

Recent evidence had received significant attention that tumor microenvironment (TME) plays a crucial role in the malignant biological behavior and progression of tumor cells (13). TME harbors cancer cells and other molecules that contribute to tumor cell growth and proliferation (14). Because of dense desmoplasia and extensive aberrant blood supply, hypoxia micrornvironment is a characteristic feature of PC (15). As a well-known hallmark of tumors, extracellular hypoxia facilitates epithelial-mesenchymal transition (EMT) *via HIF-1*α in a variety of tumors including PC (16). Surendra et al. suggested that *MUC1*-regulated *HIF-1*α stabilization mediates glucose metabolism under hypoxia microenvironment in PC (17). Emerging evidence indicated that hypoxia-induced *HIF-1*α stabilization and assessment contributes to progression and metastasis of pancreatic cancer (18). Moreover, our previous investigation indicated that *miR-646* participated in the hypoxia-induced metastasis of PC by directly regulating the expression of *MIIP*, which regulates tumor progression and *EMT* (19). Although preliminary studies on the hypoxia-induced development of PC were performed, further studies are significantly needed to focus more on novel therapies targeting the tumor hypoxia environment of PC.

Many studies have suggested that *lncRNA*s may be affected by hypoxia medium in various tumors. In colorectal carcinoma, hypoxia-induced *Lnc-LUCAT1* accelerates the *LUCAT1/PTBP1* interaction and subsequently leads to poor prognosis and worse efficiency of clinical chemotherapy (20). Li et al. showed that lncRNA *N O R A D* is significantly upregulated in hypoxia and regulate the expression of the small GTP binding protein RhoA and EMT in PC (21). Considering that the hypoxia microenvironment is also a distinctive extracellular characteristic of PC cells, we attempted to investigate whether *lncRNA*s are induced by the hypoxia microenvironment. Our previous study suggested that *lncRNA-NUTF2P3-001* is a potent tumor promoter, which is upregulated by *HIF-1*α under hypoxia microenvironment in PC (11). Nevertheless, precise regulatory mechanisms of *lncRNA*s are still not totally explored.

Histone deacetylases (HDACs) are transcriptional regulatory proteins that regulate transcription factors by drawing off acetyl groups from histones or served as posttranslational modifications (*PTMs*) which determine protein activity and stability (22, 23). Research verified that *HIF-1*α can recruit HDACs and then transcriptionally regulated target gene expression (24, 25). Previously, we revealed that *MIIP* induced acetylation of *HIF-1α* and further promotes *HIF-1α* degradation by suppressing the deacetylase ability of *HDAC6* (19). However, whether and how HDACs involved in *HIF-1α* activation during hypoxia and influenced tumor progression in PC remain unclear.

The understanding of the molecular mechanism in PC progression has greatly improved in recent decades. Zinc finger E-box-binding homeobox 1 (*ZEB1*) plays a pivotal role in cancer progression and *EMT* process, including in PC progression (26, 27). In addition, expression of *ZEB1* is induced *via* multifarious signaling pathways, including *β-catenin*, miRNA, *lncRNA*s and other factors in tumor cells (28–30).

Here, we addressed whether hypoxia-induced *HIF-1*α directly regulates *ZEB1* expression during hypoxia in PC progression. It was also suspected that *ZEB1* is essential to *HIF-1*α protein activation and potential recruitment and interaction of HDACs. In the present study, we found an upregulated *lncRNA-ZEB1-AS1* through microarray analysis for further investigation, which is approximate to *ZEB1*. More studies were further adopted to investigate reciprocal feedback on *lncRNA-ZEB1-AS1* and *HIF-1*α in PC oncogenic progression.

## Materials and Methods

### PC Patients and PC Tissue Samples

Tissue samples in this study were all gained from patients experiencing operative treatments in the First Affiliated Yijishan Hospital of Wannan Medical College (Wuhu, Anhui province, China), from May 2017 to July 2021. Our research group randomly selected 119 both PC and paired normal peritumoral (NP) tissue samples from patients without preoperative chemotherapy or radiotherapy. Clinicopathological data of patients collected in this study are all shown in **Table 1**. Operation application on those patients included pancreatectomy as well as choledochojejunostomy and gastroenterostomy, according to the criteria of the National Comprehensive Cancer Network (NCCN 2019) guideline for PC (31). Histopathology from the Department of Pathology was applied to finalize the diagnosis of PC. The samples were gathered from the PC tissues by resection or palliative surgery. A tissue biopsy gun (MG15-22, Tempe, AZ, USA) was also used to assemble PC tissues for unresectable PC patients. Next, these samples were all embedded in paraffin or stored by liquid nitrogen cryotherapy. All protocols were accepted in advance by the ethics committee of our hospital.

**Table 1:** Clinicopathological correlations of lncRNA-ZEB1-AS1 expression in pancreatic cancer.

	LncRNA-ZEB1-AS1 expression			*p*-value
	Low	High	Total	
Total cases	64	55	119	
Age (years)				
≤60	31	32	63	0.082
>60	33	23	56	
Gender				
Female	33	17	50	0.521
Male	31	38	69	
Tumor size				
≤2	25	24	49	0.054
>2	39	31	70	
Histological grade				
High/Moderate	20	23	43	0.002**
Low	44	32	76	
TNM stage				
I~II	37	28	54	0.313
III~IV	27	27	65	
Lymphatic metastasis				
Positive	40	37	77	0.001**
Negative	24	18	42	
Vascular invasion				
Positive	37	30	67	0.169
Negative	27	25	52	
Distant metastasis				
Positive	50	21	71	0.035*
Negative	14	34	48	

Overexpression of lncRNA-ZEB1-AS1 was signifcantly associated with histological grade (P = 0.002), lymphatic metastasis (P = 0.001) and distant metastasis (P = 0.035), but not with patients’ age (P = 0.082), gender (P = 0.521), tumor size (P = 0.054),TNM stage (P = 0.313) and vascular infltration P = 0.169). The p-value represents the comparison between groups (*p < 0.05, **p < 0.01).

### Cell Culture

The PC cell lines containing PANC-1, BXPC-3, AsPC-1, SW1990, and MIAPaCa-2 cell lines were all acquired from the American Type Culture Collection (ATCC, Rockville, MD, USA), while human pancreatic duct epithelial (HPDE) cells were obtained from China Beijing Be-Na Culture Collection (BNCC, Beijing, China). PC cells were authenticated and identified by DNA fingerprinting in recent 6 months, and these cell lines were passaged for less than 6 months after resuscitation. Next, we cultivated these cell lines in HyClone RPMI-1640 medium matched up with 10% characterized fetal bovine serum and penicillin (100 U/ml)–streptomycin (100 mg/ml) combination in a steady incubator of unflagging 37°C. Hypoxia simulation models were designed as our previous study (11, 12, 19), which contain two protocols: (1) hypoxia induced by fixed concentration (100 µM) of CoCl_2_ solution and (2) hypoxia induced in modular incubator chamber. The specific protocols were stated thoroughly (32).

### Transfection

Human short interfering RNAs (RNAi) for *HIF-1*α (si*HIF-1*α), *ZEB1-AS1* (si*ZEB1-AS1*), *ZEB1* (si*ZEB1*), histone deacetylase 1 (*HDAC1*) (si*HDAC1*), and negative controls (siNC) were obtained from Ribo Biological Company (Guangzhou, China). The plasmids including *HIF-1*α, *ZEB1-AS1*, *ZEB1*, and *HDAC1* and the corresponding NC plasmid were synthesized and purchased from GeneChem Company (Shanghai, China). Based on provided instructions, lipofectamine 2000 (Invitrogen, USA) were utilized for transfections. Lentivirus vector containing *ZEB1-AS1*-siRNA (LV-si*ZEB1-AS1*) and matched NC (LV-siNC) were gathered from China GeneChem Co. as well. Protein and total RNA were extracted at 48 h posttransfection after BXPC-3/PANC-1 cells were incubated in a solution with polybrene and lentivirus. Total sequences of siRNAs are exhibited on **Supplementary Table S1**.

### qRT-PCR

Total RNAs were extracted from samples or cells by means of RNAiso Plus *via* official protocols (TaKaRa, Dalian, China). All mRNAs and *lncRNA*s were reverse transcribed through the protocol of the PrimeScript^®^RT Master Mix Perfect Real Time (TaKaRa, Dalian, China) and One Step PrimeScript^®^ miRNA cDNA Synthesis Kit (Perfect Real Time) (TaKaRa, Dalian, China), following qRT-PCR analysis with the SYBR Premix Ex Taq II (TaKaRa, Dalian, China) as the official protocol. Expression levels of *lncRNA* and mRNA were measured *via* algorithm of 2^−△△^CT. This research applied GAPDH to standardize the level of *lncRNA* and mRNA expression. All qRT-PCR tests were independently tested three times. Total primers are exhibited in **Supplementary Table S2**.

### RNA Stability Assay

Pancreatic cancer cells were treated with RNA polymerase II inhibitor α-amanitin (50 μM, Sigma-Aldrich, St. Louis,MO, USA) RNAs to block new synthesis. We then collected cells at different time points (0, 6, 12, 18, and 24 h), and total RNA were extracted by using Trizol reagent (TaKaRa, Dalian, China). The levels of RNA were recorded by qRT-PCR and normalized to 18S rRNA, the percentage of the remaining RNA to test RNA degradation were measured relative to time 0.

### MTT Assay for Cell Proliferation

A proven technique of MTT assay was employed to measure the proliferation ability of PC cells. Cells were cultivated in 96-well plate (2,000/well) containing eight vices well corresponding with each sample. After cells were stably planted at 37°C, the proliferation of cells was observed and recorded from the next 1 to 5 days. For detecting the ability of proliferation of PC cells, we cultured cells by adding 20 μl MTT (5 mg/ml) to each well for 4 h. After that, we replaced the mixture with 150 μl DMSO (Sigma, USA). Following complete dissolution of the crystal in each well, ELISA reader was adopted to record the absorbance at 570 nm.

### Wound-Healing and Transwell Invasion Assays

In this study, a wound-healing assay was used to evaluate the migration capacity. We seeded PC cells into 12-well plates, and cells rise to 90% confluence manually. The cell monolayer was scratched by the tip of a micropipette and gowned in a serum-free solution. After incubation for 24 h, cell migration images were measured at 0, 24, and 48 h based on the width of the wound scratches. To explore the invasion ability, Transwell migration assays (Corning-Costar, NY, USA, pore size 8 μm) mixed with Matrigel (Sigma, USA) were applied to determine the cell invasion capacity. We placed 5 × 10^4^ PC cells, coated with 250 μl serum-free medium in the upper chamber, while the lower chamber with 700 μl solution blended with 30% FBS. Following 2 days of fixation and staining, we collected the lower chamber and further calculated the number of cells from nine fields *via* a CX33 biological microscope (Olympus, Beijing, China).

### Western Blotting and Coimmunoprecipitation

Western blot analysis has been done through repeated experiments previously (19). Protein extracts from PC cells and the concentration of protein were quantified using a BCA assay. Next, the appropriate denatured protein (30 µg per well) was separated in polyacrylamide gel electrophoresis and transferred to polyvinylidene fluoride membranes (PVDF). For this study, antibodies were recorded as follows: Rabbit anti-*ZEB1* (1:1,000, catalog#3396, Abcam, Cambridge, MA, USA), *HIF-1*α (1:1,000, catalog#279654, Abcam), *HDAC-1* (1:1,000, catalog#10197-1-AP, Proteintech, Rosemont, IL, USA), *PAN-AC* (1:1,000, catalog#A2391, ABclonal, Woburn, MA, USA), *GAPDH* (1:1,000, catalog#10494-1-AP, Proteintech). On the other hand, coimmunoprecipitation was adopted to evaluate the combining capacity on protein level. Lysates from PC cells were blended with control mouse/rabbit IgG or primary antibodies at steady-state level of 4°C throughout the night. We mingled lysates with Protein A/G PLUS-Agarose (Santa Cruz Co., Beijing, China) at 4°C for 1.5 h. Next, we separated agarose and then acquired even volumes of each sample using a lysis buffer. Western blot analysis was used for further assessments. All Western blot reactions were independently tested in triplicate.

### Chromatin immunoprecipitation

In this study, chromatin immunoprecipitation (ChIP) assay was conducted by adopting the EZ-ChIPTM kit (Millipore, Billerica, MA, USA), containing anti-*HIF-1*α and anti-RNA polymerase II antibodies (catalog#ab264350, Abcam) in accordance with manufacturer’s specification. We employed matching IgG as controls. Later, we amplified bound DNA by PCR techniques and obtained results through electrophoretic separation on a 2% agarose gel. All ChIP assays were independently tested in triplicate.

### Fluorescence *In Situ* Hybridization

For single-molecule RNA fluorescence *in situ* hybridization (FISH), we purchased The FISH Tag™ RNA Multicolor Kit (Invitrogen, USA) and MAXIscript^®^ Kit (ThermoFisher, Waltham, MA, USA). The course of experiments of RNA FISH was applied according to provided instructions (33, 34). In this study, nucleus of PC cells was stained with DAPI and red fluorescent probe was synthesized to identify *ZEB1-AS1*. Hybridization with probes was performed all night at 55°C. A laser scanning microscope (Carl Zeiss, Jena, Germany) was used for recording the stained results of FISH.

### Luciferase Reporter Assays

How *HIF-1*α modulates the binding activity of a potential hypoxia response element (HRE) on the *ZEB1-AS1* promoter during normoxia/hypoxia medium was evaluated by luciferase reporter assays. We transfected wild-type (WT) HRE sequence and mutant (MUT) HRE sequence into PANC-1 cells. Next, these cells were transfected with siNC and si*HIF-1*α which expressed pGL3-based construct involving the HRE of *ZEB1-AS1*. Dual-Luciferase Report Assay kit (Promega, Madison, WI, USA) was used for luciferase activity evaluation based on the manufacturer’s instruction (11). The intensity of the luciferase activity was standardized to Renilla luciferase. All researches were independently tested three times.

### Immunohistochemistry

Immunohistochemistry was conducted as a previously described method. Paraffin-embedded PC tissue sections from patients were collected, dried, dewaxed, and rehydrated. Sections were incubated with the primary antibody to *ZEB1* (1:1,000, catalog#3396, Abcam) or primary anti-*HIF-1*α antibody (1:1,000, catalog#279654, Abcam) and a horseradish peroxidase-conjugated secondary antibody (1:200, rabbit anti-goat). Immunohistochemical staining samples of *HIF-1*α and *ZEB1* were estimated under CX33 biological microscope (Olympus, Beijing, China). Intensity score was calculated for each sample from 0 to 3 (units of intensity) as previously described (12), and the average intensity score was further examined for each PC patient.

### Xenograft Assay

For animal experiment, we implanted PC cells, which are steadily transfected with LV-si*ZEB1-AS1*#1, LV-si*ZEB1-AS1*#2, or LV-siNC, subcutaneously in 4-week-old nude BALB/c mice based on 2 × 10^6^ cells per mouse (Beijing HFK Bio-Technology Co., Beijing, China). Five mice comprised each group and reared for 27 days. Tumor volumes (0.5 × L (length) × W^2^ (width)) were calculated every 3 days. Tumor weights were weighed after adjacent sacrifice of mice. Solid tumor tissues containing lungs and liver of the mice were excised, and we further stained the samples with H&E. The expression of *ZEB1* and *ZEB1-AS1* were calculated from five sections of metastasis tissue in each mice. Our *in vivo* experiments were approved by the Animal Research Committee of the First Affiliated Yijishan Hospital of Wannan Medical College.

### Statistical Analysis

IBM SPSS Statistics (SPSS v21.0) was applied for all analyses. Data analyses are all based on means ± SD. Specifically, group differences were evaluated by *t*-test. Paired *t*-test was employed to compare *ZEB1-AS1* in paired PC tissues. The correlation between *ZEB1-AS1* and *ZEB1* expression was measured by *Pearson’s* correlation. We used *Chi*-square test to analyze the relation between *ZEB1-AS1* and clinical characteristics. Kaplan-Meier approach was performed to describe the patients’ survivals. Further research of receiver operating characteristic curve analysis (ROC) was applied to evaluate the ability of biomarks to forecast the mortability risk of PC and area under the curve (AUC) was used to measure predictive capability (35). All assays were independently tested in triplicate. Significant differences were significant at ^*^ *p* < 0.05 and ^**^ *p* < 0.01 as marked significance.

## Results

### ZEB1-AS1 Is Overexpressed in Human PC

**Figure 1:**
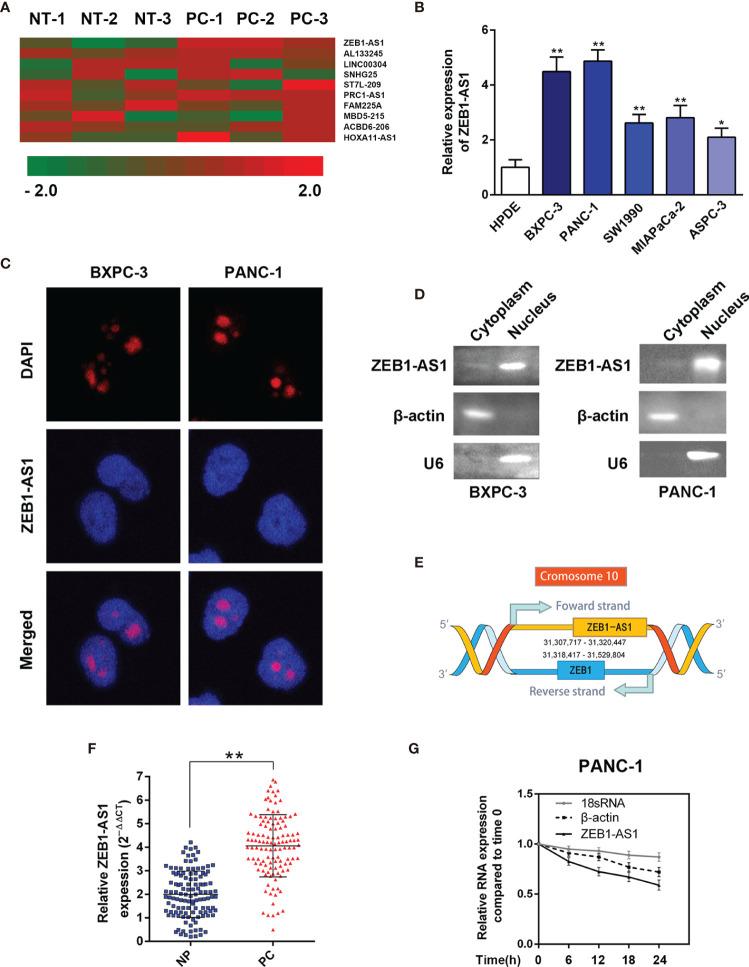
*LncRNA*-*ZEB1-AS1* expression is elevated in PC tissues and associated with PC progression. **(A)** Hierarchical clustering assessment of *lncRNA*s differentially expressed between normal pancreatic (NP) and pancreatic cancer (PC) tissues, which include *lncRNA*-*ZEB1-AS1* (*ZEB1-AS1*). **(B)** *ZEB1-AS1* expression was further compared among HPDC cells and the BxPC-3, PANC-1, SW1990, MIAPaCa, and ASPC-3 cell lines. **(C)** Single-molecule RNA FISH detection of *ZEB1-AS1* (red) in indicated PANC-1/BxPC-3 cells. DAPI-stained nuclei were blue. **(D)** The expression level of *ZEB1-AS1* in the subcellular fractions of BxPC-3 and PANC-1 cells was detected by qRT-PCR. The products of qRT-PCR were then separated by 2% agarose gel electrophoresis; U6 and β-actin were used as markers of the nucleus and cytoplasm, respectively. **(E)** Genomic location of *ZEB1-AS1* and *ZEB1* with transcript orientation marked by arrows. **(F)** *ZEB1-AS1* expression was assessed by qRT-PCR in 119 PC and paired NP tissues. **(G)** RNA synthesis in PANC-1 cells was blocked by α-amanitin (50 µM), and *ZEB1-AS1* stability was assessed by qRT-PCR compared with 0 h RNA polymerases II and I were used to transcribe β-actin and 18S rRNA, respectively. All data were presented as means ± SD of at least three independent experiments. Values are significant at ^*^ *p* < 0.05 and ^**^ *p* < 0.01 as indicated.

To determine the underlying dysregulated *lncRNA*s that induce the tumorigenesis of PC, we analyzed a *lncRNA* microarray. Compared with paired noncancerous peritumoral (NP) tissues, hierarchical clustering data demonstrated that *lncRNA-ZEB1-AS1* (*ZEB1-AS1*) was one of the significantly upregulated *lncRNA*s in PC samples (**Figure 1A**). Compared with the normal HPDE cell line, the expression of *ZEB1-AS1* in five PC cell lines (BXPC-3, PANC-1, SW1990, MIAPaCa-2, and AsPC-1) was significantly higher (**Figure 1B**). Moreover, FISH analysis was performed to determine whether *ZEB1-AS1* is located in the nucleus or cytoplasm of BXPC-3/PANC-1 cells. It was indicated that *ZEB1-AS1* was mainly located in the nucleus (**Figure 1C**). Although *ZEB1-AS1* was a well-known oncogene in many tumors, there has been little research on PC. Hence, it was selected for further investigation in consideration of the genomic location on *ZEB1* and *ZEB1-AS1* (**Figure 1D**). The full-length *ZEB1-AS1* sequence was determined, and the secondary structure of *ZEB1-AS1* was determined with minimum free energy (MFE) by searching the *NONCODE* online database (**Supplementary Figures S1A, B**). Online tools, including the Coding Potential Assessment Tool (CPAT) and Coding Potential Calculator (CPC), predicted that *ZEB1-AS1* is a potential noncoding RNA (**Supplementary Figures S1C, D**).

To further verify the microarray results, *ZEB1-AS1* levels were measured in 119 paired PC and NP tissues using real-time PCR analysis. The *ZEB1-AS1* transcript levels in the PC tissues were significantly higher than those in paired noncancerous tissues (**Figure 1E**). After blocking RNA synthesis through the RNA polymerase II inhibitor, α-amanitin, in PANC-1 cells, the levels of *ZEB1-AS1* were evaluated. The results indicate that α-amanitin treatment decreased *ZEB1-AS1* level significantly after 24 h, instead of the level of 18S mRNA. The results verified that *ZEB1-AS1* synthesis was regulated by RNA polymerase II rather than RNA polymerase I (**Figure 1F**).

### ZEB1-AS1 Promotes Proliferation Ability and Invasion Capacity in PC Cells

**Figure 2:**
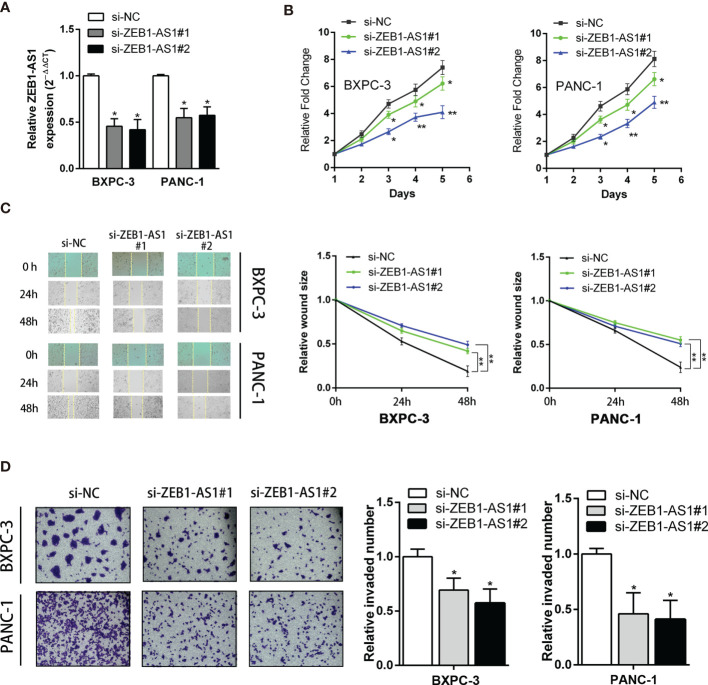
Knockdown of ZB1-AS1 inhibited proliferation, migration, and invasion of PC cells. **(A)** *ZEB1*AS1 inhibiting efficiency in BxPC-3 and PANC-1 cells were analyzed by qRT-PCR. **(B)** Proliferation of BxPC-3/PANC-1 cells underexpressing *ZEB1-AS1* or negative control was assessed *via* MTT assays for 5 days. **(C)** Migration ability was evaluated by wound-healing assay. Representative images (left) and relative wound size are shown (right). **(D)** Transwell assay (left) was designed to assess the invasion ability of those transfected BxPC-3/PANC-1 cells. Average counts of five random microscopic fields were analyzed. The histogram (right) shows the percentage of invaded PC cell number. All data were presented as means ± SD of at least three independent experiments. Values are significant at ^*^ *p* < 0.05 and ^**^ *p* < 0.01 as indicated.

Because *ZEB1-AS1* is upregulated in PC tissues, short interference siRNAs for *ZEB1-AS1* (si*ZEB1-AS1*) were utilized to suppress the levels of *ZEB1-AS1* in PC cells. Two si*ZEB1-AS1* siRNAs targeting *ZEB1-AS1* were designed, and they significantly knocked down endogenous *ZEB1-AS1* expression and were used for further experiments (**Figure 2A**). To elucidate potential impacts of *ZEB1-AS1*, we transfected si*ZEB1-AS1* and a *ZEB1-AS1* overexpression (*ZEB1-AS1*-OE) vector into BXPC-3 and PANC-1 cells. Knockdown of *ZEB1-AS1* suppressed the proliferation and migration of BXPC-3 and PANC-1 cell lines (**Figures 2B, C**). In addition, Matrigel-coated Transwells confirmed that *ZEB1-AS1* depletion caused a significant inhibition of the invasion ability of BXPC-3 and PANC-1 cells (**Figure 2D**). Furthermore, transfection of *ZEB1-AS1*-OE into BXPC-3 and PANC-1 cells resulted in overexpression of *ZEB1-AS1* compared with the vector control (**Supplementary Figure S2A**). Moreover, overexpression of *ZEB1-AS1* markedly induced the proliferation, migration, and invasion capacity of these cells (**Supplementary Figures S2B–D**). Taken together, these results demonstrated that *ZEB1-AS1* promotes the proliferation capacity and invasion ability of PC cells.

### ZEB1 Is a Key Target of ZEB1-AS1 Implementing the Biological Action of PC Cells Under Hypoxia Conditions

**Figure 3:**
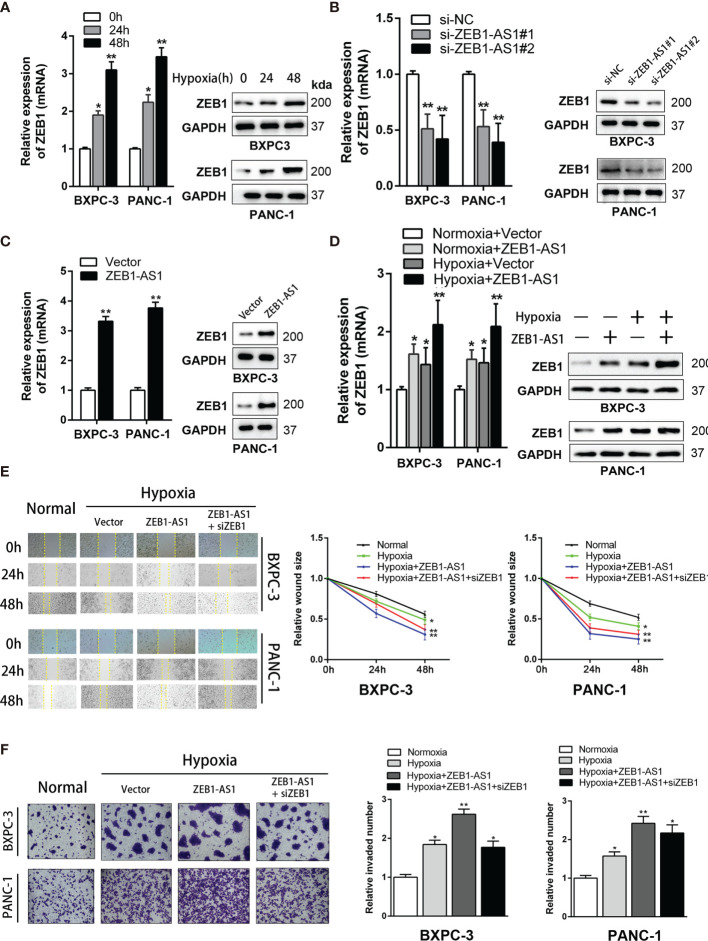
*ZEB1* is a critical target of *ZEB1-AS1* exerting functions in PC cells during hypoxia condition. **(A)** Expression of *ZEB1* in BxPC-3/PANC-1 cells cultured with hypoxia medium for 0, 24, and 48 h were analyzed at the mRNA (left) and protein (right) levels by qRT-PCR and Western blot analysis, respectively. **(B)** Expression of *ZEB1* in BxPC-3/PANC-1 cells transfected with *ZEB1-AS1* siRNAs (si-*ZEB1-AS1*#1, si-*ZEB1-AS1*#2) or the negative control siRNA (siNC) was analyzed at the mRNA (left) and protein (right) levels by qRT-PCR and Western blot analysis, respectively. **(C)** Expression of *ZEB1* in BxPC-3/PANC-1 cells transfected with *ZEB1-AS1* overexpression plasmid (*ZEB1-AS1*) or the empty vector plasmid (Vector) was analyzed at the mRNA (left) and protein (right) levels by qRT-PCR and Western blot analysis, respectively. **(D)** *ZEB1* in *ZEB1-AS1* overexpressing BxPC-3/PANC-1 cells cultured in normal or hypoxia condition was analyzed at the mRNA and protein levels by qRT-PCR and Western blot analysis, respectively. **(E, F)** Migration and invasion ability of four groups of BxPC-3/PANC-1 cells were recorded by the Wound healing assay and Transwell assay, representative images were shown (left). Average counts of five random microscopic fields were analyzed. The histogram (right) shows the percentage of migrated or invaded PC cells number. All data were presented as means ± SD of at least three independent experiments. Values are significant at ^*^ *p* < 0.05 and ^**^ *p* < 0.01 as indicated.

Many studies have established that *lncRNA*s modulate the levels of neighboring genes by *cis* regulation. As *ZEB1-AS1* is near the *ZEB1* gene, which is a tumorigenic driver in several cancers, we hypothesized that *ZEB1-AS1* exerts a *cis*-acting role on *ZEB1* expression. The expression of *ZEB1* increased with prolonged culture in a hypoxia microenvironment from 0 to 48 h (**Figure 3A**). However, the expression quantity of *ZEB1* under normoxia conditions from 0 to 48 h of culture did not show a significant difference (**Supplementary Figure S3A**). In BXPC-3 and PANC-1 cells, *ZEB1-AS1* knockdown significantly decreased *ZEB1* mRNA and protein expression (**Figure 3B**). It is inspiring to note that overexpression of *ZEB1-AS1* showed a similar trend (**Figure 3C**).

Hypoxia medium (hypoxia microenvironment undergoing 48 h) was used for further experiments. In this study, hypoxia medium increases the mRNA and protein levels of *ZEB1-AS1*-induced *ZEB1* expression (**Figure 3D**), indicating that *ZEB1* is a potential target of *ZEB1-AS1* under hypoxia conditions. Similarly, the invasion and migration capacities of BXPC-3 and PANC-1 cells were upregulated in *ZEB1-AS1*-overexpressing cells under hypoxia conditions, and these effects were weakened by si-*ZEB1* (**Figures 3E, F**). Together, these results demonstrated that *ZEB1* is critical for *ZEB1-AS1* to exert its oncogenic and malignant functions in PC cells under hypoxia conditions.

### ZEB1-AS1 Is Modulated Transcriptionally by HIF-1α in PC Cells Under Hypoxia Conditions

**Figure 4:**
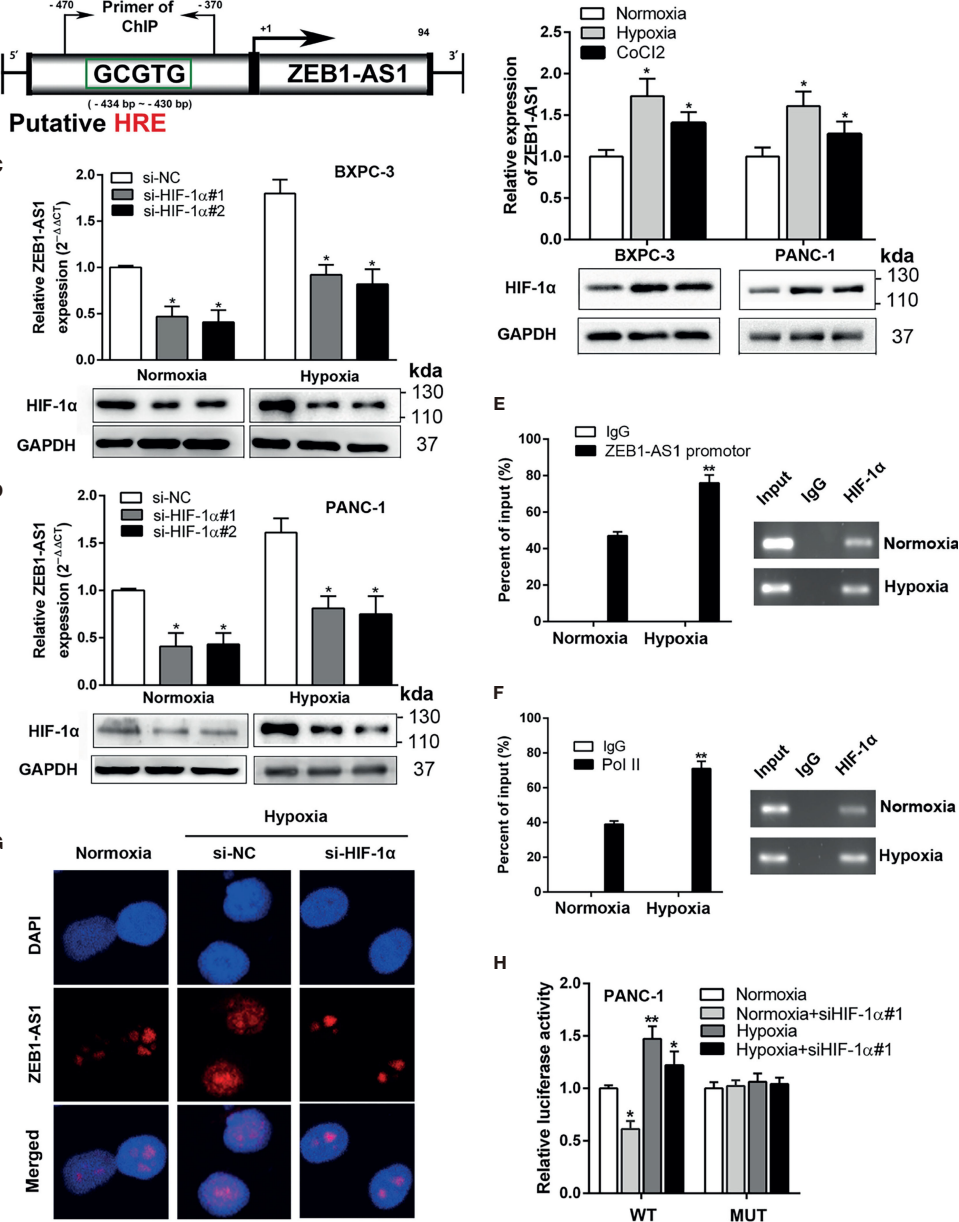
ZEB-1AS1 transcription is regulated by *HIF-1*α during hypoxia medium. **(A)** A putative hypoxia-responsive element (HRE) was found in the promoter region of *ZEB1-AS1*. **(B)** The expression levels of *ZEB1-AS1* (upper) and *HIF-1*α protein (lower) in BXPC-3/PANC-1 cells were measured after being cultured during normoxia, hypoxia (1%O_2_), or CoCl_2_ (concentration of 100 µM under 48 h) at the mRNA and protein levels by qRT-PCR and Western blot analysis, respectively. **(C, D)** After knockdown of *HIF-1*α with siRNA, the expression of *ZEB1-AS1* was evaluated by qRT-PCR in BXPC-3 and PANC-1 cells under normoxia or hypoxia (upper). Lower diagrams indicated *HIF-1*α protein levels by Western blot analysis. **(E)** ChIP assays with anti-*HIF-1*α antibody were performed to affirm the binding between *HIF-1*α and HRE of *ZEB1-AS1* promoter region in PANC-1 cells under normoxia or hypoxia condition. **(F)** After being cultured in hypoxia or normoxia, ChIP assays with anti-Pol II antibody were performed to ascertain the binding capacity between Pol II and *ZEB1-AS1* promoter region in PANC-1 cells. **(G)** After knockdown of *HIF-1*α, the expression of *ZEB1-AS1* was shown by FISH assays in PANC-1 cells during normoxia and hypoxia condition. **(H)** Wild-type *ZEB1-AS1* promoter-containing *pGL3* reporter vector (WT) or mutant-type *ZEB1-AS1* promoter-containing *pGL3* reporter vector (MUT) of *ZEB1-AS1* promoter sequence firefly luciferase reporter activity in PANC-1 cells transfected with siNC or si*HIF-1*α and cultured under normoxia or hypoxia conditions were assessed by Renilla luciferase reporter assays after 48 h All data were presented as means ± SD of at least three independent experiments. Values are significant at ^*^ *p* < 0.05 and ^**^ *p* < 0.01 as indicated.

Next, we investigated the potential mechanism by which hypoxia conditions control *ZEB1-AS1* expression in PC cells. Recent studies have investigated whether variable expression of *lncRNA*s is attributed to hypoxia conditions in a variety of cancer cells. Because our previous research indicated that hypoxia facilitates *lncRNA-NUTF2P3* enrichment (11), we investigated whether transcriptionally upregulated *ZEB1-AS1* is closely related to the hypoxia environment of PC. Genomic structure analysis indicated one predictable HRE in the promoter region of *ZEB1-AS1*, suggesting that *ZEB1-AS1* is a hypoxia-responsive *lncRNA* (**Figure 4A**). The levels of *ZEB1-AS1* also statistically increased under hypoxia medium but are insignificantly different under normoxia environment in the same time course (from 0 to 48 h) of the experiment (**Supplementary Figures S3B, C**).

After treatment with hypoxia medium or chemically inducing hypoxia with CoCl_2_ (100 µM) for 48 h, the level of *ZEB1-AS1*, in accordance with *HIF-1*α expression, was significantly increased in BXPC-3 and PANC-1 cells (**Figure 4B**). Moreover, *HIF-1*α knockdown suppressed hypoxia-induced *ZEB1-AS1* upregulation in PC (**Figures 4C, D**). ChIP assays showed that the HRE region in the *ZEB1-AS1* promoter modulated *HIF-1*α binding to the *ZEB1-AS1* promoter area, which was further increased under hypoxia conditions (**Figure 4E**). Pol-II was also shown to bind to the HRE of the *ZEB1-AS1* promoter region. Similarly, this binding was enhanced during hypoxia medium, indicating a protranscriptional ability of this binding (**Figure 4F**). Furthermore, RNA-FISH assay demonstrated that hypoxia-induced *ZEB1-AS1* overexpression was suppressed through *HIF-1*α knockdown (**Figure 4G**). To further verify the functional binding of *HIF-1*α to the *ZEB1-AS1* promoter region, we performed a luciferase reporter assay in PANC-1 cells, which were transfected with luciferase reporter vectors containing the WT promoter of *ZEB1-AS1* or MUT promoter of *ZEB1-AS1*. Hypoxia medium remarkably increased the luciferase activity in cells transfected with WT but not MUT *ZEB1-AS1*. However, knockdown of *HIF-1*α decreased the hypoxia-induced luciferase activity of PC cells transfected with WT *ZEB1-AS1* (**Figure 4H**). Thus, these findings suggested that *ZEB1-AS1* is transcriptionally regulated by *HIF-1*α in PC cells in hypoxia medium.

### ZEB1-AS1 Stabilizes HIF-1α Protein Through ZEB1-Mediated Deacetylation

**Figure 5:**
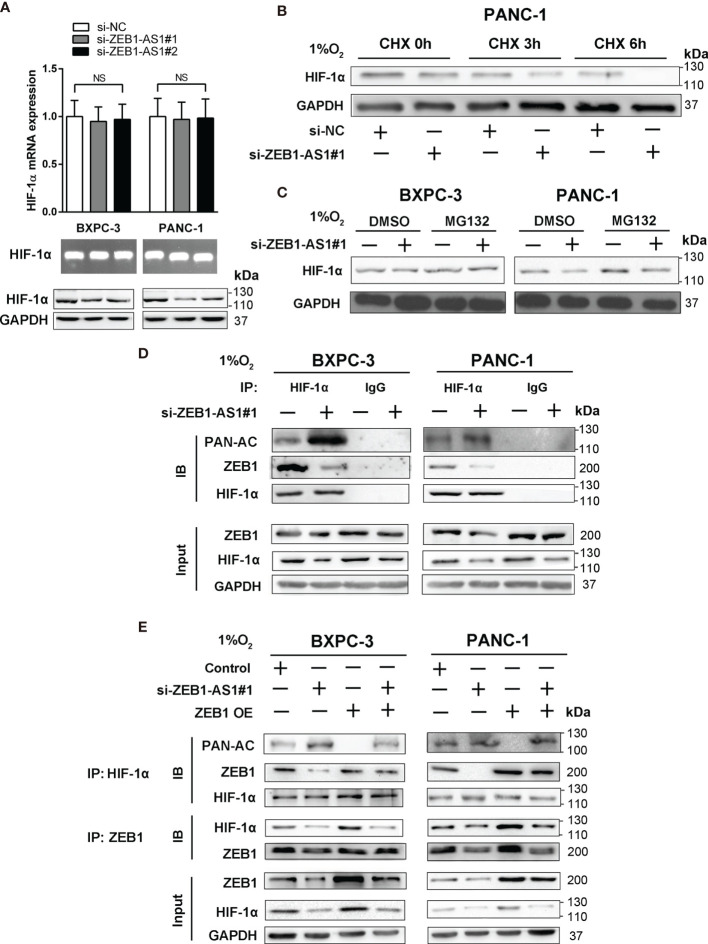
Hypoxia-induced *ZEB1-AS1* stabilizes *HIF-1*α by promoting deacetylation. **(A)** *HIF-1*α mRNA and protein levels were analyzed by qRT-PCR (upper), 2% agarose gel electrophoresis (middle), and Western blot (lower) analysis in *ZEB1-AS1*-knockdown BxPC-3/PANC-1 cells. **(B)** The effect of *ZEB1-AS1* knockdown on stabilization of *HIF-1*α was measured in PANC-1 cells treated with cycloheximide (CHX) under hypoxia conditions of three time points (0, 3, and 6 h). **(C)** *HIF-1*α levels in the *ZEB1-AS1*-knockdown BxPC-3/PANC-1 cells were analyzed under hypoxia condition with or without MG132 treatment. **(D)** *HIF-1*α acetylation and the binding between *HIF-1*α and *ZEB1* in the *ZEB1-AS1*-knockdown BxPC-3/PANC-1 cells under hypoxia condition for 24 h were analyzed in the cell lysates following antiacetylation, anti-*HIF-1*α, or anti-*ZEB1* immunoprecipitation. **(E)** The acetylation of *HIF-1*α and the binding between *HIF-1*α and *ZEB1* in the BxPC-3/SW1990 cells, cultured under hypoxia condition, transfected with si*ZEB1-AS1* or/and *ZEB1*-OE were analyzed by immunoprecipitation with antiacetylation, anti-*HIF-1*α, or anti-*ZEB1* antibody. All data were presented as means ± SD of at least three independent experiments. NS means the difference is not significant.

The relationship between *ZEB1-AS1* and *HIF-1*α was further explored to determine the mechanism by which *ZEB1-AS1* affects the interaction between *HIF-1*α and the *ZEB1* promoter. The results showed that the protein level of *HIF-1*α was suppressed by the knockdown of *ZEB1-AS1*, indicating that *HIF-1*α may be a direct target of *ZEB1-AS1* in BXPC-3 and PANC-1 cells. However, downregulation of *ZEB1-AS1* did not affect the mRNA expression of *HIF-1*α, and a similar experimental result was repeated by RNA agarose gel electrophoresis (**Figure 5A**). These findings indicated that *ZEB1-AS1* influenced the level of *HIF-1*α at the posttranscriptional level and not at the transcriptional level. Furthermore, we investigated how *ZEB1-AS1* regulates the protein stability of *HIF-1*α. The *HIF-1*α protein level was measured following cycloheximide (CHX) treatment, which disrupts protein synthesis. During CHX treatment, the stability of *HIF-1*α was inhibited *via* downregulation of *ZEB1-AS1* in a time-dependent manner (**Figure 5B**). Next, MG132 (proteasome inhibitor) was used to rescue the decrease in *HIF-1*α protein levels in *ZEB1-AS1* knockdown PC cells (**Figure 5C**). We further verified that *ZEB1-AS1* knockdown significantly induced *HIF-1*α acetylation and decreased the *ZEB1* and *HIF-1*α binding under hypoxia conditions (**Figure 5D**). Moreover, we demonstrated that *ZEB1* overexpression rescued the *ZEB1-AS1* knockdown-induced downregulation of *HIF-1*α and decreased *HIF-1*α and *ZEB1* binding, and it reduced the acetylation level of *HIF-1*α (**Figure 5E**).

*HDAC1* interacts with *HIF-1*α and decreases the protein level of *HIF-1*α. Research has shown that the transcriptional repressor, *ZEB1*, recruits *HDAC1*-containing corepressor complexes (CRC) to decrease transcription of the cadherin 1 (*CDH1*) gene and downregulate E-cadherin in PC. Thus, we hypothesized that *HDAC1* participates in *ZEB1-AS1*-promoted destabilization of *HIF-1*α in PC cells. Coimmunoprecipitation (CoIP) demonstrated that *HIF-1*α and *HDAC1* bind to each other and that this binding is intensified by hypoxia conditions (**Supplementary Figure S4A**). This result indicated that *HDAC1*, *HIF-1*α, and *ZEB1* may form a *ZEB1-AS1*-induced complex. To investigate whether *HDAC1* is involved in the regulation of *HIF-1*α protein expression by *ZEB1*, we used trichostatin A (TSA), a specific inhibitor of HDACs, to assess the stability of *HIF-1*α protein. The results showed that TSA aggravated the inhibitory effect of *ZEB1* knockdown on *HIF-1*α protein levels under hypoxia conditions in PC (**Supplementary Figure S4B**). Consistently, TSA downregulated the upregulation of *HIF-1*α at the protein level, which was promoted by *ZEB1* overexpression (**Supplementary Figure S4C**). In addition, overexpression of *HDAC1* rescued the suppression of *HIF-1*α expression by *ZEB1* knockdown. However, *HDAC1* knockdown inhibited *HIF-1*α expression, which was upregulated by *ZEB1* overexpression (**Supplementary Figures S5A, B**). To evaluate the effect of *HDAC1* on the interaction of *HIF-1*α with *ZEB1*, we measured the acetylated levels of *HIF-1*α *via* a CoIP assay. The results revealed that *ZEB1* repressed TSA-induced *HIF-1*α acetylation (**Supplementary Figure S5C**). In contrast, TSA-induced si-*ZEB1* accelerated *HIF-1*α protein acetylation (**Supplementary Figure S5D**). Together, these results suggested that *ZEB1-AS1* promotes an interaction among *HDAC1*, *HIF-1*α, and *ZEB1*, which inhibits acetylation of *HIF-1*α by promoting the deacetylase capacity of *HDAC1*, further resulting in *HIF-1*α stabilization.

### Depletion of ZEB1-AS1 Inhibits the Progression and Metastasis of PC *In Vivo*

**Figure 6:**
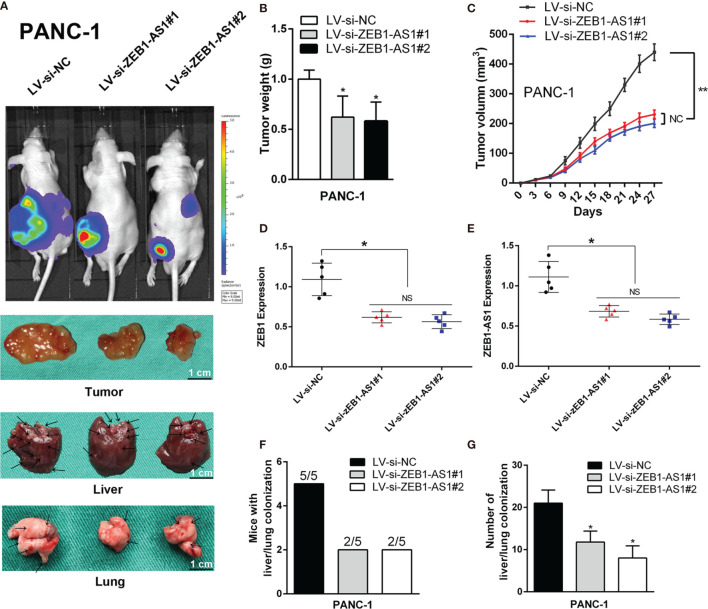
*ZEB1-AS1* facilitates the *in vivo* invasion and proliferation of PANC-1 cells. PANC-1 cells were stably transfected with lentivirus containing si-*ZEB1-AS1* sequence. Transfected cells were injected subcutaneously into the right flank of 4-week-old male BALB/c nude mice. **(A)** After stably transfected, mice were sacrificed after 4 weeks. The fluorescence imaging *in vivo* demonstrated the subcutaneous tumor, liver, and lung invasion nodules. Arrows indicate the invasion nodules. Scale bars, 1 cm. **(B**, **C)** Tumor weight and volume in LV-siNC, LV-si-*ZEB1-AS1*#1, and LV-si-*ZEB1-AS1*#2 groups. The tumor volumes were calculated every 3 days (tumor volume = 0.5 × length × width^2^). **(D**, **E)** *ZEB1-AS1* and *ZEB1* expression was detected in the subcutaneous tumor from LV-si-*ZEB1-AS1*#1, LV-si-*ZEB1-AS1*#2, or LV-siNC group. **(F)** Liver metastasis was measured with the indicated PANC-1 cells. *N* = 5 mice in each group. **(G)** The number of visible liver metastases per five sections in each nude mouse. All data were presented as means ± SD of at least three independent experiments. The arrows showed the invasion nodules. Values are significant at ^*^ *p* < 0.05 and ^**^ *p* < 0.01 as indicated. NS means the difference is not significant.

To further evaluate the role of *ZEB1-AS1*, PANC-1 cells stably transfected with a lentiviral vector including a NC sequence (LV-siNC) or *ZEB1-AS1*-siRNA sequences (LV-siB*ZEB1-AS1*#1 or LV-siB*ZEB1-AS1*#2) were implanted into 4-week-old nude mice. Compared with the LV-siNC group, the tumor size of the LV-si*ZEB1-AS1* groups was smaller. Simultaneously, the visible number of liver or lung metastases were lower in the LV-si*ZEB1-AS1* groups (**Figure 6A**), and the tumor growth rate and weight of the LV-si*ZEB1-AS1* groups were suppressed (**Figures 6B, C**). qRT-PCR analysis showed that the transcription levels of *ZEB1-AS1* and *ZEB1* in the LV-si*ZEB1-AS1* group were significantly lower than those of the LV-siNC group (**Figures 6D, E**). Next, we found that both the number of mice with lung or liver metastases and the number of distinct metastatic nodes in the LV-si*ZEB1-AS1* groups were significantly reduced compared with the control mice (**Figures 6F, G**). We further verified the above results *via* H&E staining and further detected the RNA expression quantity of *ZEB1* and *ZEB1-AS1* in lung or liver metastases (**Supplementary Figure S6**). Similarly, LV-Si*ZEB1-AS1* suppressed the proliferation and metastasis ability of BXPC-3 cells *in vivo* (**Supplementary Figures S7** and **S8**). Overall, these results confirmed the tumorigenic mechanism of *ZEB1-AS1* in PC cells.

### The ZEB1-AS1/ZEB1 Feedback Loop Is Correlated With PC Prognosis

**Figure 7:**
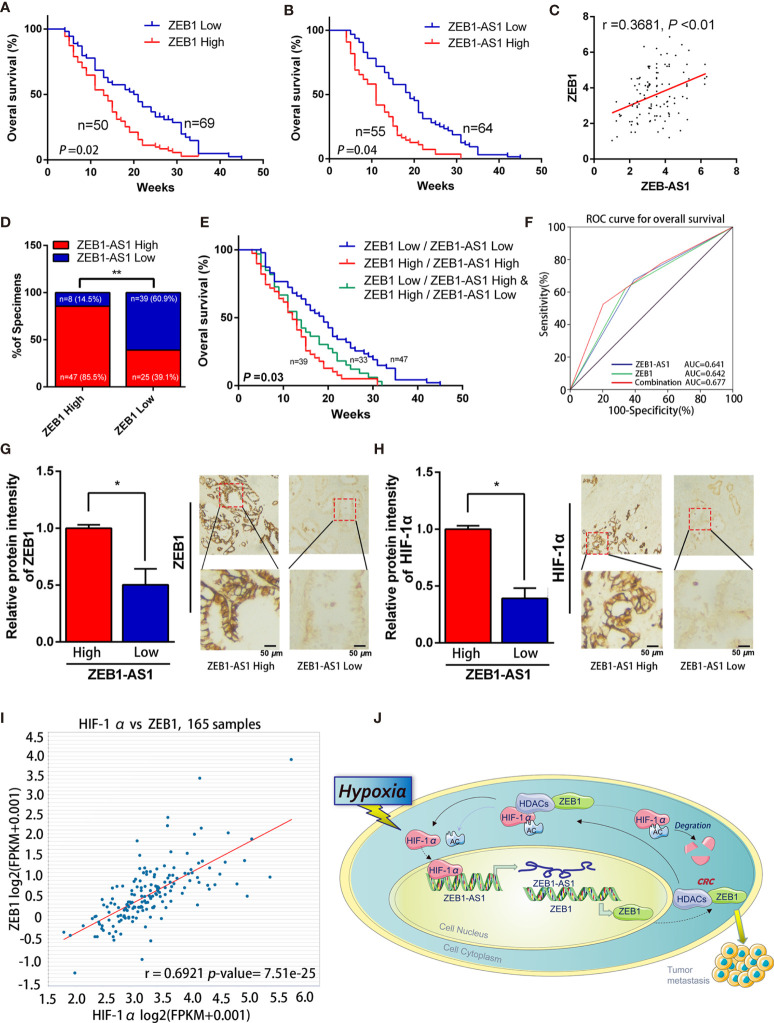
*ZEB1-AS1* was upregulated in PC and correlates with poor clinical outcome. **(A)** Overall survival of 119 patients with PC was analyzed by generation of Kaplan-Meier curves. The low or high *ZEB1* groups are below or above the 50th percentile of *ZEB1* expression, respectively. **(B)** Overall survival of 119 patients with PC was analyzed by generation of Kaplan-Meier curves. The low or high *ZEB1-AS1* groups are below or above the 50th percentile of *ZEB1-AS1* expression, respectively. **(C)** Pearson’s correlation analysis was applied to confirm the relationship of the expression between *ZEB1-AS1* and *ZEB1* in 119 patients with PC. **(D)** Correlation between *ZEB1-AS1* and *ZEB1* in specimens of patients with PC were analyzed by Chi-square test. **(E)** Kaplan-Meier analysis of overall survival for patients with 119 PC patients based on the number of upregulated molecular markers including *ZEB1-AS1* and *ZEB1*. Patients were divided into three groups based on the expression of *ZEB1-AS1* and *ZEB1* at RNA levels. **(F)** ROC curve analysis for overall survival for *ZEB1-AS1* (AUC = 0.641 (95% CI, 0.536–0.746), *p* = 0.012), *ZEB1* (AUC = 0.642 (95% CI, 0.536–0.748), *p* = 0.012) as individual biomarkers or for the combined panel (AUC = 0.677 (95% CI, 0.572–0.781), *p* = 0.002). Area under the curve (AUC) was measured to assess predictive capability; 95% CI, 95% confidence interval. **(G, H)** *ZEB1* and *HIF-1*α expression in high or low levels of *ZEB1-AS1* from tissues of PC patients analyzed by IHC. Histogram showed the relative protein intensity *via* IHC scores (left). Representative images of stained sections were confirmed (right). **(I)** Correlation of *ZEB1* and *HIF-1*α RNA expression was analyzed from the TCGA pancreatic cancer dataset through applying online database ChIPBase. **(J)** A graphical representation of the proposed mechanisms, hypoxia induced *ZEB1-AS1* expression which promotes *ZEB1* upregulation and then further cause malignant progression of PC cells.

The clinicopathological results showed that the overexpression level of *ZEB1-AS1* was positively associated with histological grade, lymphatic invasion, and distant metastasis in PC patients (**Table 1**). Furthermore, Kaplan-Meier analysis revealed that PC patients with high expression of *ZEB1* experienced a shorter overall survival (OS) time than those patients with low *ZEB1* expression (**Figure 7A**). The Kaplan-Meier study also demonstrated a correlation between higher *ZEB1-AS1* mRNA expression and shorter OS (**Figure 7B**). In addition, we found that *ZEB1-AS1* and *ZEB1* expression suggested a positive relationship in 119 patients with PC, which was assessed *via* Pearson’s correlation analysis or Chi-square test (**Figures 7C, D**). Moreover, the survival analysis indicated that low expression levels of both *ZEB1* and *ZEB1-AS1* were most beneficial to the OS of patients followed by the high expression level of either *ZEB1* or *ZEB1-AS1*, while overexpression levels of both *ZEB1* and *ZEB1-AS1* had the worst effect (**Figure 7E**). We further performed a ROC curve analysis of the predictive value for OS. The results confirmed that the combination of *ZEB1* and *ZEB1-AS1* showed a preferable additive value (**Figure 7F**). Immunohistochemical (IHC) assays demonstrated that the protein expression of both *ZEB1* and *HIF-1*α positively correlated with the expression levels of *ZEB1-AS1* (**Figures 7G, H**). TCGA data from ChIPBase V2.0 (36) revealed that *HIF-1*α expression had a positive relationship with *ZEB1* levels in PC (**Figure 7I**). Therefore, these results suggested that *HIF-1*α/*ZEB1-AS1*/*ZEB1*/*HDAC1* signaling is involved in the oncogenesis and metastasis of PC (**Figure 7J**).

## Discussion

Although thousands of *lncRNA*s have been explored, the full functional mechanism of most molecules is still unclear, especially in pancreatic cancer. Many studies have reported that *ZEB1-AS1* correlates with bladder cancer, prostate cancer, gastric cancer, and colorectal cancer (37–40). Nonetheless, the connection between *ZEB1-AS1* and PC is rarely reported. In addition, overexpression of *ZEB1-AS1* is related to malignant characteristics and short overall survival (41). In PC patients, high expression of *ZEB1-AS1* was usually positively related to histological grade, TNM stage, lymphatic invasion, vascular invasion, distant metastasis, and short overall survival time (42). Thus, these data demonstrated that *ZEB1-AS1* may promote oncogenesis and metastasis of PC.

As *ZEB1-AS1* is adjacent to *ZEB1*, which is a metastasis-related protein in various tumors (43), *ZEB1* is a transcription factor that induces EMT and plays a crucial role in the progression of DNA damage, cancer cell differentiation, metastasis, and chemoresistance in human cancers (26, 44, 45). It is well known that *ZEB1-AS1* is an antisense *lncRNA*, which is derived from the promoter region of *ZEB1*. *ZEB1-AS1* induced *ZEB1* expression in direct or indirect way in different cancers, and it is demonstrated to be closely related to the unfavorable prognosis of malignant tumors such as prostatic carcinoma (38), glioma (46), and hepatocellular carcinoma (47). However, Liu et al. reported that *ZEB1* contrarily inhibited *ZEB1-AS1* expressions in osteosarcoma cell (48). Now, this leads us to ask: how *ZEB1-AS1* regulates *ZEB1* in PC unprecedentedly?

The present study suspected weather the *ZEB1* was regulated by *ZEB1-AS1* at the transcriptional level in PC, and we wonder if it is possible to establish a positive relationship between *ZEB1* and *ZEB1-AS1*. Targeting the *lncRNA*/*ZEB1* pathway may be a potential strategy for clinical treatments of PC.

According to recent studies, the hypoxia microenvironment of PC, owing to unlimited proliferation of tumor cells and aberrant blood supply, which increase oxygen consumption and generate a characteristic feature of the microenvironment of solid tumors (49). Compared with the areas of well-oxygenated tumors, hypoxia areas within PC are closely associated with tumor malignancy and with poor prognosis (50). Under hypoxia pressure, hypoxia-inducible factors (HIFs) is a chief regulator, which are composed of unstable subunits (*HIF-α* and *HIF-β*) and accumulates to abet cell resistance to temporary stress (51). Additionally, signaling routing among hypoxia-induced HIFs facilitate aggressive malignancy and treatment resistance in PC cells. Although studies of the relationship between hypoxia conditions and metastasis in PC are still in the early stage, hypoxia microenvironment has great potential as a target of pancreatic cancer to enhance tumor therapies in the future.

Recent studies have demonstrated that *lncRNA*s are affected under hypoxia conditions and are regulated by *HIF-1*α to a great extent. For example, *lncRNA RP11* is promoted by hypoxia/*HIF-1*α and is vital for metastasis and EMT (52). Song et al. reported that hypoxia-induced *lncRNA-AC020978* facilitates tumorigenesis and glycolytic metabolism *via* the *PKM2*/*HIF-1*α axis in nonsmall cell lung cancer (53). Similarly, our previous studies suggested that *HIF-1*α-induced *lncRNA NUTF2P3-001* reduces *miR-3923/KRAS* expression, leading to metastasis of PC (11). In reaction to hypoxia, *HIF-1*α, which indicates *HIF-1* activity, plays a major role in regulating downstream target genes (54). This active transcription factor binds to the HRE in the promotor region, thereby inducing downstream gene expression (55). Because the gene sequence analysis was available in the NCBI database, we are prone to collect potential HRE location in the promoter of *ZEB1-AS1*. Thus, it takes further experiments to prove the theory and to demonstrate the possibility of a pathway for hypoxia-induced *ZEB1* regulation in PC, which is probably mediated by *ZEB1-AS1*.

In our previous study (12), we revealed that *MTA2TR* promotes the protein stability of *HIF-1*α through *MTA2*-promoted deacetylation. However, the potential regulatory mechanism of *HIF-1*α deacetylation is still unknown. Recent studies have verified that *HIF-1*α stability is modulated through *VHL* ubiquitination complex-mediated proteasomal degradation (56) and confirmed that *HIF-1*α stability is further managed by acetylation, which is regulated *via* the *ARD1* gene (57) or the HDACs (58). In addition, our previous reports indicated that *MIIP* is an anticancer biomarker for the oncogenesis of PC, and we also affirmed that *MIIP* increases acetylation and induces *HIF-1*α protein degradation, which is reversed by *HDAC6* overexpression (19).

As *ZEB1-AS1* regulates *ZEB1* expression, we investigated whether *ZEB1-AS1* is involved in the protein stabilization of *HIF-1*α in hypoxia medium. Schneider et al. demonstrated that the *ZEB1*-*HDAC* axis is involved in EMT process (59). *ZEB1* is a repressor of the *CDH1* gene in pancreatic cancer, and the *CDH1* gene is epigenetically silenced during the dynamic EMT process. One crucial pathway involves the transcriptional repressor, *ZEB1*, which recruits *HDAC1*- and/or *HDAC2*-containing CRCs to suppress transcription of the *CDH1* gene. Aghdassi et al. also indicated that HDAC inhibitors or *ZEB1* knockdown plays a role in antiproliferative and antimigratory characteristics in PC cells (60). In addition, our previous study demonstrated that *HIF-1*α/*HADC1* transcriptionally restricts the expression of *miR-548an* under hypoxia conditions, leading to the progression of PC (61). We speculated that the potential connection among *ZEB1*, HDACs, and *HIF-1*α may regulate *HIF-1*α stability by modulating HDAC activity. Thus, these results strongly suggested that *ZEB1* inhibits acetylation of *HIF-1*α by promoting the deacetylation activity of HDACs, thereby accelerating *HIF-1*α stabilization.

In conclusion, the present study demonstrated the complex involvement of the *HIF-1*α/*ZEB1-AS1*/*ZEB1* pathway in adjusting the cellular response to the hypoxia environment of PC. Our findings suggested that *ZEB1-AS1* may be a biological target for the clinical detection and treatment of PC.

## Data Availability Statement

The datasets presented in this study can be found in online repositories. The names of the repository/repositories and accession number(s) can be found in the article/**Supplementary Material**.

## Ethics Statement

The studies involving human participants were reviewed and approved by the Ethics Committee of Wannan Medical College. The patients/participants provided their written informed consent to participate in this study. The animal study was reviewed and approved by the Institutional Committee on Animal Care of Wannan Medical College.

## Author Contributions

YJ, ZMZ, and QY conceived and coordinated the study and drafted and revised the manuscript. XH and QK performed the cell culture and some cellular experiments. HH and ZZ performed the molecular experiments. YJ, YX, and HS analyzed the data. All authors contributed to the article and approved the submitted version.

## Funding

The authors would like to acknowledge the grant support from the National Natural Science Foundation of China (81902515) and the University Natural Science Research Project of Anhui Province (KJ2019A0429 and KJ2019A0412).

## Conflict of Interest

The authors declare that the research was conducted in the absence of any commercial or financial relationships that could be construed as a potential conflict of interest.

## Publisher’s Note

All claims expressed in this article are solely those of the authors and do not necessarily represent those of their affiliated organizations, or those of the publisher, the editors and the reviewers. Any product that may be evaluated in this article, or claim that may be made by its manufacturer, is not guaranteed or endorsed by the publisher.

## Supplementary Material

Supplementary Figure 1 The sequence, secondary structure and coding capacity of *ZEB1-AS1*. **(A)** The sequence and gene loci of *lncRNA-ZEB1-AS1*. **(B)** Prediction of *ZEB1-AS1* secondary structure according to minimum free energy (MFE) and relative partition function from RNAfold web server. **(C, D)** The transcript’s noncoding nature was figured out by coding potential assessment tool (CPAT) and coding potential calculator (CPC). Results suggested that *ZEB1-AS1* is more likely to be a noncoding sequence than a coding one.

Supplementary Figure 2
*ZEB1-AS1* overexpression impedes the proliferation and invasion in PC cell. **(A)** *ZEB1-AS1* expression was recorded *via* qRT-PCR after the BXPC-3/PANC-1 cells transfected with a negative control plasmid (NC) or one encoding human full-length *ZEB1-AS1*. **(B)** Power of Proliferation in BXPC-3/PANC-1 cells, which overexpressing *ZEB1-AS1* or control were detected by MTT assays for 5 days. **(C)** Migration capacity was evaluated *via* wound healing assay. Representative graphics (left) and relative wound size were analyzed (right). **(D)** Transwell assay (left) was designed to investigate the invasion extent of those transfected BXPC-3/PANC-1 cells. Mean values of five random micro areas were analyzed. The proportion of invaded PC cells number was measured through histogram (right). Data processing and statistical analysis used SPSS21.0 statistical analysis package and quantitative data were manifested as means ± SD of at least three experiments independently. Values with statistic difference are significant at **P* < 0.05 and ***P* < 0.01 as marked significance.

Supplementary Figure 3 The expression of *ZEB1* and *ZEB1-AS1* under normoxia/hypoxia conditions from 0 - 48 hours of culture. **(A)** Expression of *ZEB1* in BxPC-3/PANC-1 cells cultured with normoxia medium (0 h, 24 h and 48 h) were evaluated at the mRNA (left) and protein (right) levels by qRT-PCR and Western blot analysis, respectively. **(B, C)** Indeed, we observed that under hypoxia conditions the levels of ZEB1-AS1 statistically increase when compared with control. However, it does not change under normoxia conditions. Data processing and statistical analysis used SPSS21.0 statistical analysis package and quantitative data were manifested as means ± SD of at least three experiments independently. Values with statistic difference are significant at **P* < 0.05 and ***P* < 0.01 as marked significance.

Supplementary Figure 4
*HDAC1* is called for *ZEB1*-mediated *HIF-1*α deacetylation during hypoxia. **(A)** After PANC-1 cells were cultivated during normoxia/hypoxia medium, Co-IP was executed by the usage of an anti–*HIF-1*α or anti-*HDAC1* antibody. The protein levels of *HIF-1*α and *HDAC1* were certified *via* western blot analysis. **(B)** Following the treatment during hypoxia, protein level of *HIF-1*α in PANC-1 cells treated with histone deacetylase inhibitor - trichostatin A (TSA) and (or) *ZEB1* inhibition (si-*ZEB1*) were recorded by WB. **(C)** WB analysis manifested plasmid pcDNA3-*ZEB1* (*ZEB1*) reinforced *HIF-1α* level under hypoxia, which was reverted *via* TSA. Data processing and statistical analysis used SPSS21.0 statistical analysis package and quantitative data were manifested as means ± SD of at least three experiments independently. Values with statistic difference are significant at **P* < 0.05 and ***P* < 0.01 as marked significance.

Supplementary Figure 5
*ZEB1* promotes deacetylation of *HIF-1*α *via* inhibiting acetylase capacity of *HDAC1*. **(A)** After treatment during hypoxia medium, the expression of *HIF-1*α was detected *via* WB analysis of PANC-1 cells transfected with pcDNA3-*HDAC1* and (or) *ZEB1* inhibition (si-*ZEB1*). **(B)** *HDAC1* knockdown restrained *HIF-1*α expression, which was increased by plasmid pcDNA3-*ZEB1* (*ZEB1*). **(C)** Following treated with MG132, analysis of Co-IP was conducted applying an anti-*HIF-1*α, anti-acetylation in PANC-1 cells treated with pcDNA3-*ZEB1* (*ZEB1*) or TSA. Levels of *HIF-1*α, acetylated *HIF-1*α proteins were calculated by WB analysis. **(D)** Co-IP displayed that the TSA-induced *HIF-1*α acetylation was enhanced by *ZEB1* inhibition (si-*ZEB1*) through using MG132. GAPDH played the role of endogenous control. All data were presented at lowst three independent experiments.

Supplementary Figure 6
*ZEB1-AS1* promotes the PC metastasis of BXPC-3 cells in mice. From the point of the pathology, H&E images of liver extracted from LV-si-*ZEB1-AS1*#1, LV-si-*ZEB1-AS1*#2 or LV-siNC group. Scale bars, 100 μm. **(B)** RNA expression quantity of *ZEB1* and *ZEB1-AS1* were measured from the liver metastases. **(C)** Applying H&E-stained images for description of lung tissue extracted from LV-si-*ZEB1-AS1*#1, LV-si-*ZEB1-AS1*#2 or LV-siNC group. The scale bars displayed 100 μm in the diagrams. **(D)** RNA expression level of *ZEB1* and *ZEB1-AS1* were detected from the lung metastases. quantitative data were manifested as means ± SD of at least three experiments independently. Values with statistic difference are significant at *P < 0.05 and **P < 0.01 as marked significance.

Supplementary Figure 7
*ZEB1-AS1* promotes the *in vivo* invasion ability and proliferation capacity of BXPC-3 cells. Following si-*ZEB1-AS1* sequence lentivirus were stably transfected in PANC-1 cells, we injected transfected BXPC-3 cells into the 4-week-old nude mice (male BALB/c) at right flank subcutaneously. **(A)** After stably transfected and 4 weeks feeding, mice were sacrificed for further experiments. The fluorescence imaging *in vivo* demonstrated the subcutaneous tumor, liver and lung invasion nodules. Arrows showed the invasion nodules. Scale bars is 1 cm in the diagrams. **(B, C)** Tumor weight and volume in LV-siNC, LV-si-*ZEB1-AS1*#1 and LV-si- *ZEB1-AS1*#2 groups. We recorded and calculated the tumor volumes every 3 days *via* the equation: volume = 0.5×length×width^2^). **(D, E)** The expression of *ZEB1-AS1* and *ZEB1* were perceived in the subcutaneous tumor from LV-si-*ZEB1-AS1*#1, LV-si-*ZEB1-AS1*#2 or LV-siNC group. **(F)** Liver and lung metastasis was recorded of the indicated BXPC-3 cells. Each group contained 5 mice in this study. **(G)** The number of visible liver/lung metastases was measured in mouse. Data processing and statistical analysis used SPSS21.0 statistical analysis package and quantitative data were manifested as means ± SD of at least three experiments independently. Values with statistic difference are significant at *P < 0.05 and **P < 0.01 as marked significance.

Supplementary Figure 8
*ZEB1-AS1* promotes the PC metastasis of PANC-1 cells in mice. H&E images of liver extracted from LV-si-*ZEB1-AS1*#1, LV-si-*ZEB1-AS1*#2 or LV-siNC group of PANC-1 cells in mice. Scale bars, 100 μm. **(B)** The *ZEB1* and *ZEB1-AS1* expression were measured *via* qRT-PCR from the liver metastases. **(C)** Detecting H&E-stained images for lung tissue extracted from LV-si-*ZEB1-AS1*#1, LV-si-*ZEB1-AS1*#2 or LV-siNC group. The scale bars displayed 100 μm in the diagrams. **(D)** RNA expression level of *ZEB1* and *ZEB1-AS1* were evaluated from the lung metastases. quantitative data were manifested as means ± SD of at least three experiments independently. Values with statistic difference are significant at *P < 0.05 and **P < 0.01 as marked significance.
