# Corrigendum: Positive Reciprocal Feedback of LncRNA ZEB1-AS1 and HIF-1α Contributes to Hypoxia–Promoted Tumorigenesis and Metastasis of Pancreatic Cancer

**DOI:** 10.3389/fonc.2021.821077

**Published:** 2021-12-07

**Authors:** Yan Jin, Zhengming Zhang, Qiao Yu, Zhu Zeng, Hong Song, Xiaoxu Huang, Qi Kong, Hao Hu, Yabin Xia

**Affiliations:** ^1^ Department of Gastrointestinal Surgery, The First Affiliated Yijishan Hospital of Wannan Medical College, Wuhu, China; ^2^ Department of Gastroenterology, Union Hospital, Tongji Medical College, Huazhong University of Science and Technology, Wuhan, China; ^3^ Department of Emergency Surgery, Union Hospital, Tongji Medical College, Huazhong University of Science and Technology, Wuhan, China; ^4^ Department of Pathology, The First Affiliated Yijishan Hospital of Wannan Medical College, Wuhu, China

**Keywords:** pancreatic cancer, *lncRNA-ZEB1-AS1*, *Zeb1*, *HIF-1α*, metastasis, hypoxia

In the original article, there was a mistake in **Figure 4** as published. The western blot of reference gene (GAPDH) in hypoxia group in **Figure 4D** was incorrectly used and plotted in this figure. However, this mistake doesn’t affect the results of experiment and understanding of our research on purpose. The corrected **Figure 4** appears below.

**Figure 4:**
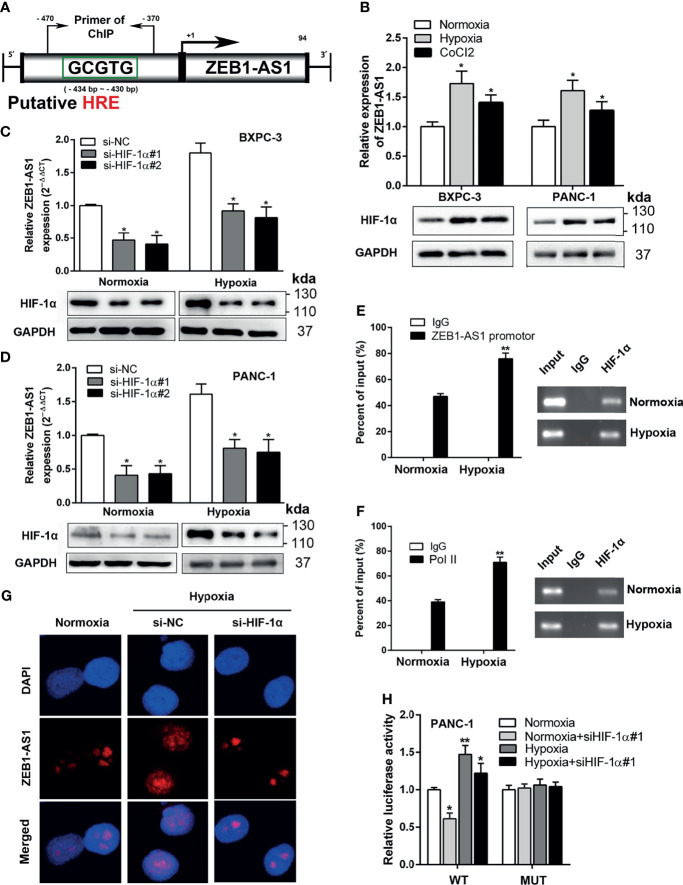
ZEB-1AS1 transcription is regulated by HIF-1α during hypoxia medium. **(A)** A putative hypoxia-responsive element (HRE) was found in the promoter region of ZEB1-AS1. **(B)** The expression levels of ZEB1-AS1 (upper) and HIF-1α protein (lower) in BXPC-3/PANC-1 cells were measured after being cultured during normoxia, hypoxia (1%O2), or CoCl2 (concentration of 100 µM under 48 h) at the mRNA and protein levels by qRT-PCR and Western blot analysis, respectively. **(C, D)** After knockdown of HIF-1α with siRNA, the expression of ZEB1-AS1 was evaluated by qRT-PCR in BXPC-3 and PANC-1 cells under normoxia or hypoxia (upper). Lower diagrams indicated HIF-1α protein levels by Western blot analysis. **(E)** ChIP assays with anti-HIF-1α antibody were performed to affirm the binding between HIF-1α and HRE of ZEB1-AS1 promoter region in PANC-1 cells under normoxia or hypoxia condition. **(F)** After being cultured in hypoxia or normoxia, ChIP assays with anti-Pol II antibody were performed to ascertain the binding capacity between Pol II and ZEB1-AS1 promoter region in PANC-1 cells. **(G)** After knockdown of HIF-1α, the expression of ZEB1-AS1 was shown by FISH assays in PANC-1 cells during normoxia and hypoxia condition. **(H)** Wild-type ZEB1-AS1 promoter-containing pGL3 reporter vector (WT) or mutant-type ZEB1-AS1 promoter-containing pGL3 reporter vector (MUT) of ZEB1-AS1 promoter sequence firefly luciferase reporter activity in PANC-1 cells transfected with siNC or siHIF-1α and cultured under normoxia or hypoxia conditions were assessed by Renilla luciferase reporter assays after 48 h All data were presented as means ± SD of at least three independent experiments. Values are significant at *p ≤ 0.05 and **p ≤ 0.01 as indicated.

The authors apologize for this error and state that this does not change the scientific conclusions of the article in any way. The original article has been updated.

## Publisher’s Note

All claims expressed in this article are solely those of the authors and do not necessarily represent those of their affiliated organizations, or those of the publisher, the editors and the reviewers. Any product that may be evaluated in this article, or claim that may be made by its manufacturer, is not guaranteed or endorsed by the publisher.

