# Supplementary figures and images for: Positive Reciprocal Feedback of lncRNA ZEB1-AS1 and HIF-1α Contributes to Hypoxia-Promoted Tumorigenesis and Metastasis of Pancreatic Cancer
Source: Front Oncol. 2021 Nov 22;11:761979. doi: 10.3389/fonc.2021.761979 (PMC8645903; doi:10.3389/fonc.2021.761979)

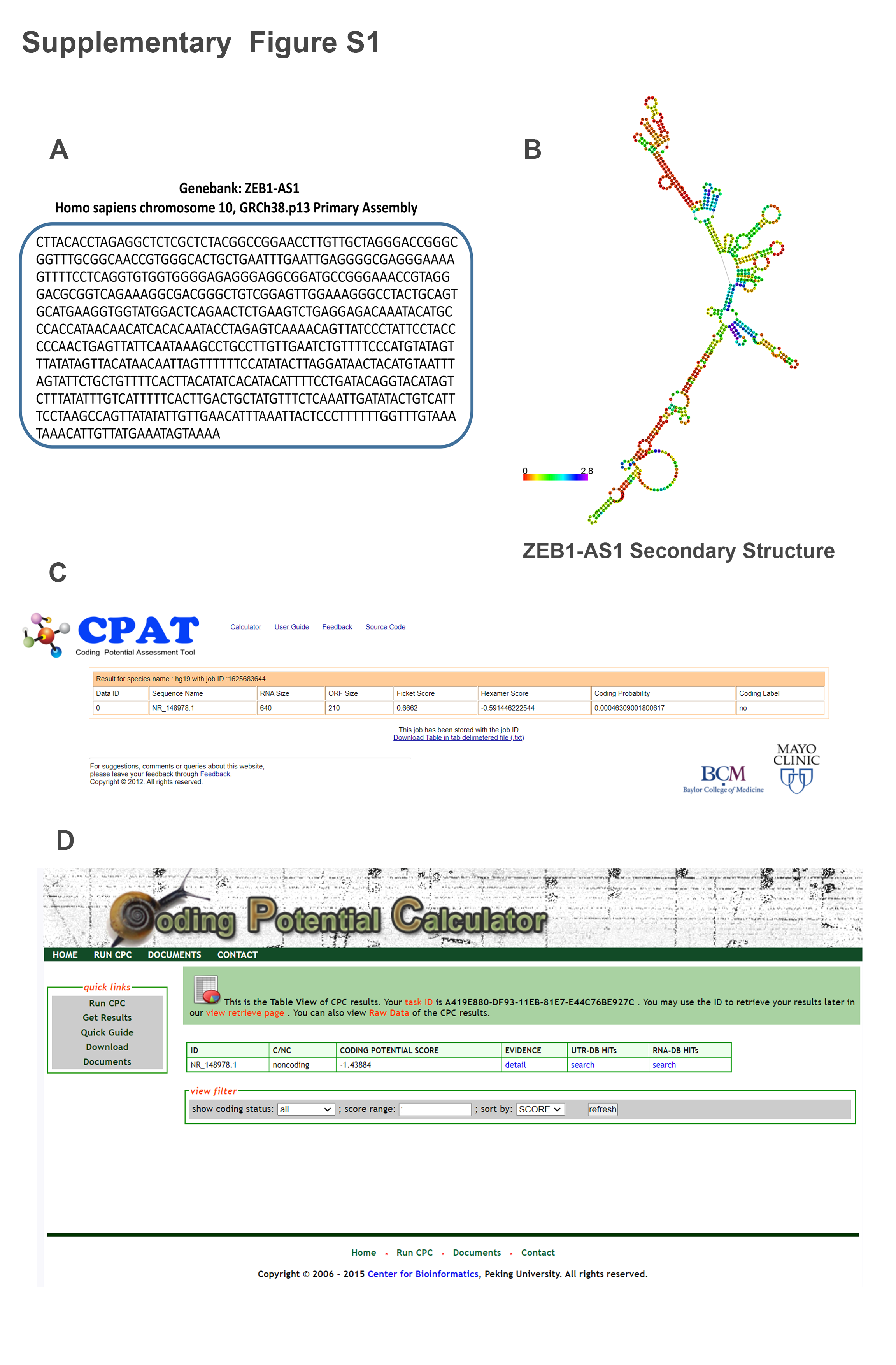

Supplement: Supplementary Figure 1 — The sequence, secondary structure and coding capacity of ZEB1-AS1. (A) The sequence and gene loci of lncRNA-ZEB1-AS1. (B) Prediction of ZEB1-AS1 secondary structure according to minimum free energy (MFE) and relative partition function from RNAfold web server. (C, D) The transcript’s noncoding nature was figured out by coding potential assessment tool (CPAT) and coding potential calculator (CPC). Results suggested that ZEB1-AS1 is more likely to be a noncoding sequence than a coding one. [file Image_1.tif]

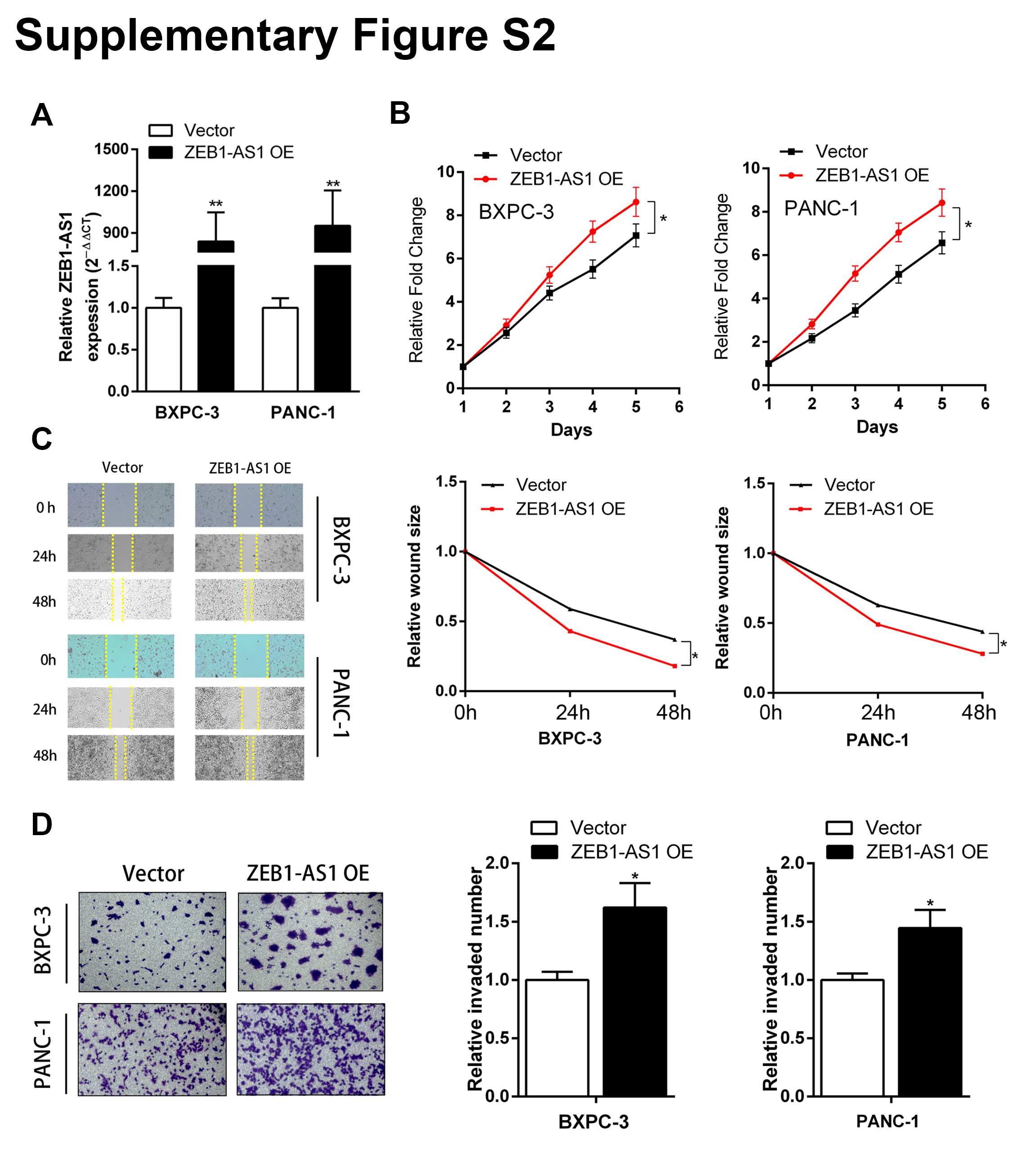

Supplement: Supplementary Figure 2 — ZEB1-AS1 overexpression impedes the proliferation and invasion in PC cell. (A) ZEB1-AS1 expression was recorded via qRT-PCR after the BXPC-3/PANC-1 cells transfected with a negative control plasmid (NC) or one encoding human full-length ZEB1-AS1. (B) Power of Proliferation in BXPC-3/PANC-1 cells, which overexpressing ZEB1-AS1 or control were detected by MTT assays for 5 days. (C) Migration capacity was evaluated via wound healing assay. Representative graphics (left) and relative wound size were analyzed (right). (D) Transwell assay (left) was designed to investigate the invasion extent of those transfected BXPC-3/PANC-1 cells. Mean values of five random micro areas were analyzed. The proportion of invaded PC cells number was measured through histogram (right). Data processing and statistical analysis used SPSS21.0 statistical analysis package and quantitative data were manifested as means ± SD of at least three experiments independently. Values with statistic difference are significant at *P < 0.05 and **P < 0.01 as marked significance. [file Image_2.tif]

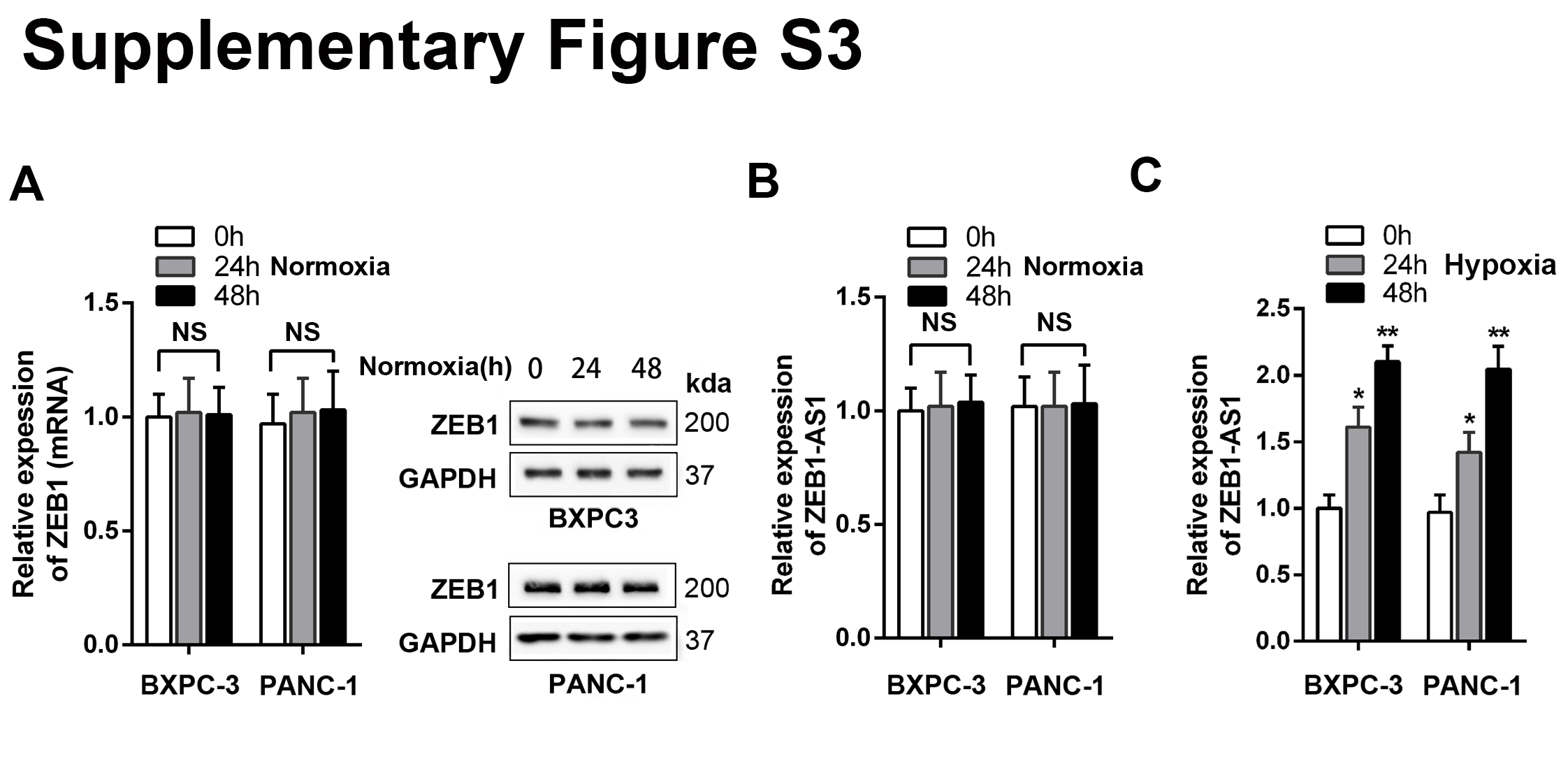

Supplement: Supplementary Figure 3 — The expression of ZEB1 and ZEB1-AS1 under normoxia/hypoxia conditions from 0 - 48 hours of culture. (A) Expression of ZEB1 in BxPC-3/PANC-1 cells cultured with normoxia medium (0 h, 24 h and 48 h) were evaluated at the mRNA (left) and protein (right) levels by qRT-PCR and Western blot analysis, respectively. (B, C) Indeed, we observed that under hypoxia conditions the levels of ZEB1-AS1 statistically increase when compared with control. However, it does not change under normoxia conditions. Data processing and statistical analysis used SPSS21.0 statistical analysis package and quantitative data were manifested as means ± SD of at least three experiments independently. Values with statistic difference are significant at *P < 0.05 and **P < 0.01 as marked significance. [file Image_3.tif]

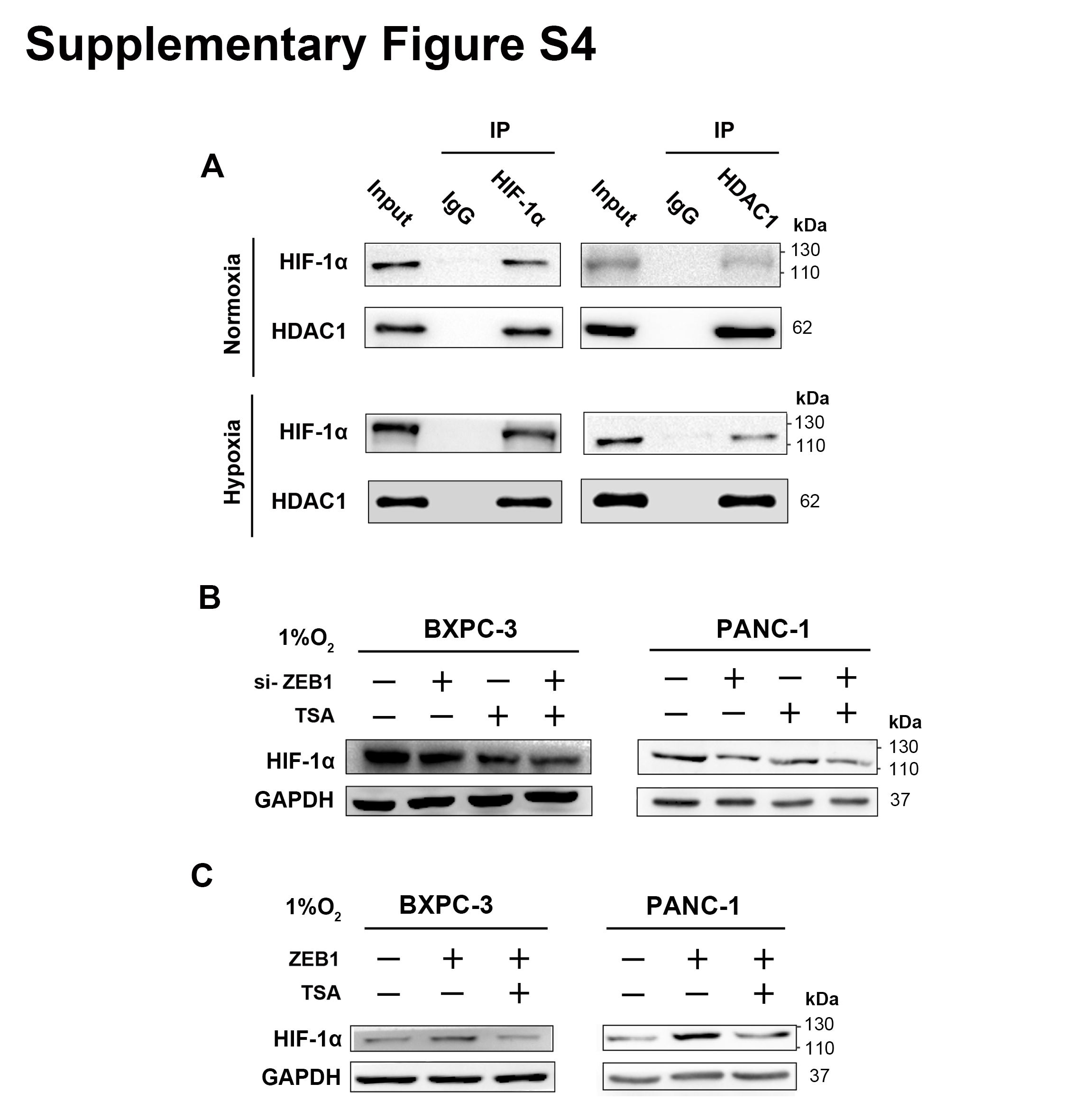

Supplement: Supplementary Figure 4 — HDAC1 is called for ZEB1-mediated HIF-1α deacetylation during hypoxia. (A) After PANC-1 cells were cultivated during normoxia/hypoxia medium, Co-IP was executed by the usage of an anti–HIF-1α or anti-HDAC1 antibody. The protein levels of HIF-1α and HDAC1 were certified via western blot analysis. (B) Following the treatment during hypoxia, protein level of HIF-1α in PANC-1 cells treated with histone deacetylase inhibitor - trichostatin A (TSA) and (or) ZEB1 inhibition (si-ZEB1) were recorded by WB. (C) WB analysis manifested plasmid pcDNA3-ZEB1 (ZEB1) reinforced HIF-1α level under hypoxia, which was reverted via TSA. Data processing and statistical analysis used SPSS21.0 statistical analysis package and quantitative data were manifested as means ± SD of at least three experiments independently. Values with statistic difference are significant at *P < 0.05 and **P < 0.01 as marked significance. [file Image_4.tif]

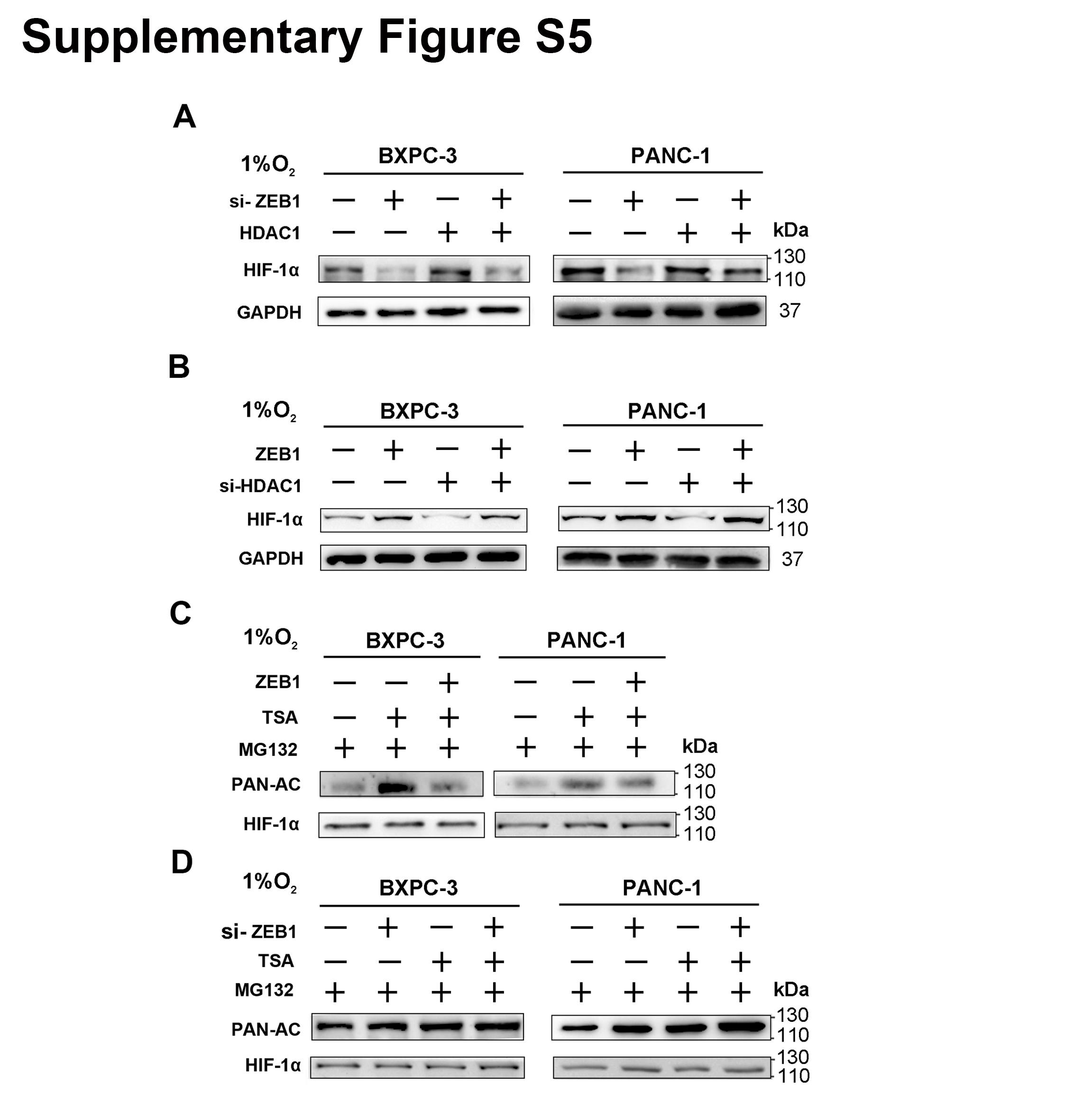

Supplement: Supplementary Figure 5 — ZEB1 promotes deacetylation of HIF-1α via inhibiting acetylase capacity of HDAC1. (A) After treatment during hypoxia medium, the expression of HIF-1α was detected via WB analysis of PANC-1 cells transfected with pcDNA3-HDAC1 and (or) ZEB1 inhibition (si-ZEB1). (B) HDAC1 knockdown restrained HIF-1α expression, which was increased by plasmid pcDNA3-ZEB1 (ZEB1). (C) Following treated with MG132, analysis of Co-IP was conducted applying an anti-HIF-1α, anti-acetylation in PANC-1 cells treated with pcDNA3-ZEB1 (ZEB1) or TSA. Levels of HIF-1α, acetylated HIF-1α proteins were calculated by WB analysis. (D) Co-IP displayed that the TSA-induced HIF-1α acetylation was enhanced by ZEB1 inhibition (si-ZEB1) through using MG132. GAPDH played the role of endogenous control. All data were presented at lowst three independent experiments. [file Image_5.tif]

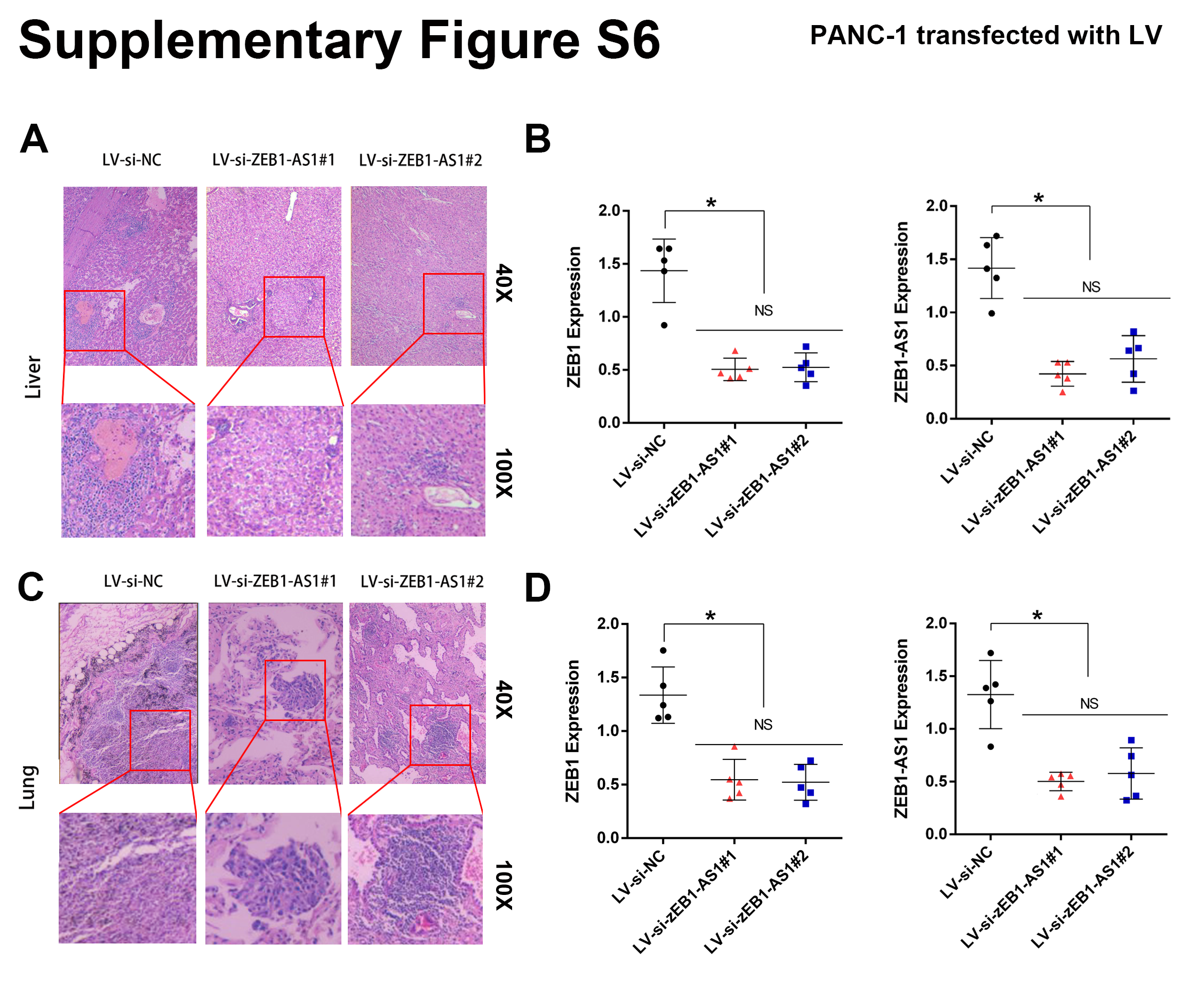

Supplement: Supplementary Figure 6 — ZEB1-AS1 promotes the PC metastasis of BXPC-3 cells in mice. From the point of the pathology, H&E images of liver extracted from LV-si-ZEB1-AS1#1, LV-si-ZEB1-AS1#2 or LV-siNC group. Scale bars, 100 μm. (B) RNA expression quantity of ZEB1 and ZEB1-AS1 were measured from the liver metastases. (C) Applying H&E-stained images for description of lung tissue extracted from LV-si-ZEB1-AS1#1, LV-si-ZEB1-AS1#2 or LV-siNC group. The scale bars displayed 100 μm in the diagrams. (D) RNA expression level of ZEB1 and ZEB1-AS1 were detected from the lung metastases. quantitative data were manifested as means ± SD of at least three experiments independently. Values with statistic difference are significant at *P < 0.05 and **P < 0.01 as marked significance. [file Image_6.tif]

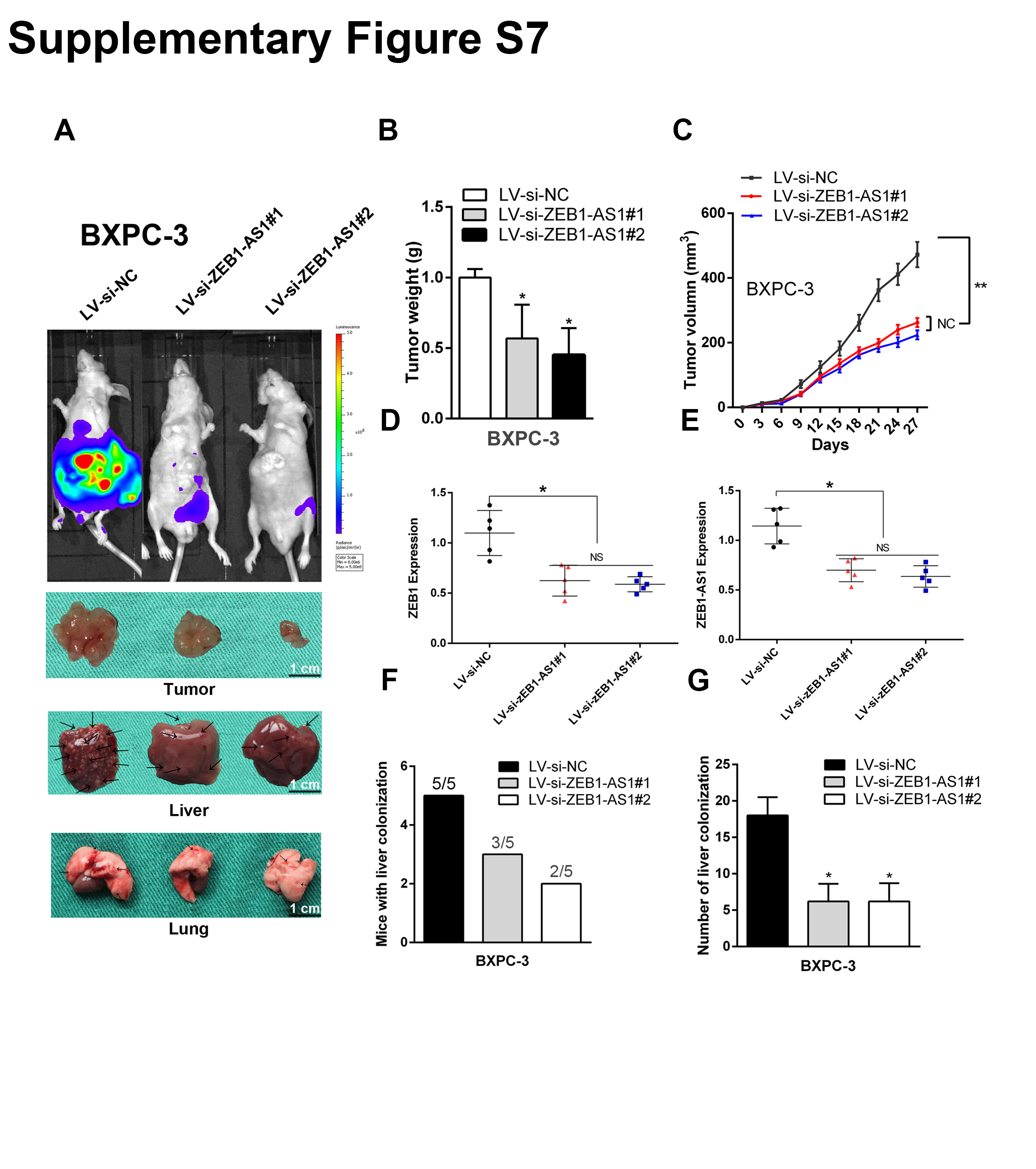

Supplement: Supplementary Figure 7 — ZEB1-AS1 promotes the in vivo invasion ability and proliferation capacity of BXPC-3 cells. Following si-ZEB1-AS1 sequence lentivirus were stably transfected in PANC-1 cells, we injected transfected BXPC-3 cells into the 4-week-old nude mice (male BALB/c) at right flank subcutaneously. (A) After stably transfected and 4 weeks feeding, mice were sacrificed for further experiments. The fluorescence imaging in vivo demonstrated the subcutaneous tumor, liver and lung invasion nodules. Arrows showed the invasion nodules. Scale bars is 1 cm in the diagrams. (B, C) Tumor weight and volume in LV-siNC, LV-si-ZEB1-AS1#1 and LV-si- ZEB1-AS1#2 groups. We recorded and calculated the tumor volumes every 3 days via the equation: volume = 0.5×length×width2). (D, E) The expression of ZEB1-AS1 and ZEB1 were perceived in the subcutaneous tumor from LV-si-ZEB1-AS1#1, LV-si-ZEB1-AS1#2 or LV-siNC group. (F) Liver and lung metastasis was recorded of the indicated BXPC-3 cells. Each group contained 5 mice in this study. (G) The number of visible liver/lung metastases was measured in mouse. Data processing and statistical analysis used SPSS21.0 statistical analysis package and quantitative data were manifested as means ± SD of at least three experiments independently. Values with statistic difference are significant at *P < 0.05 and **P < 0.01 as marked significance. [file Image_7.tif]

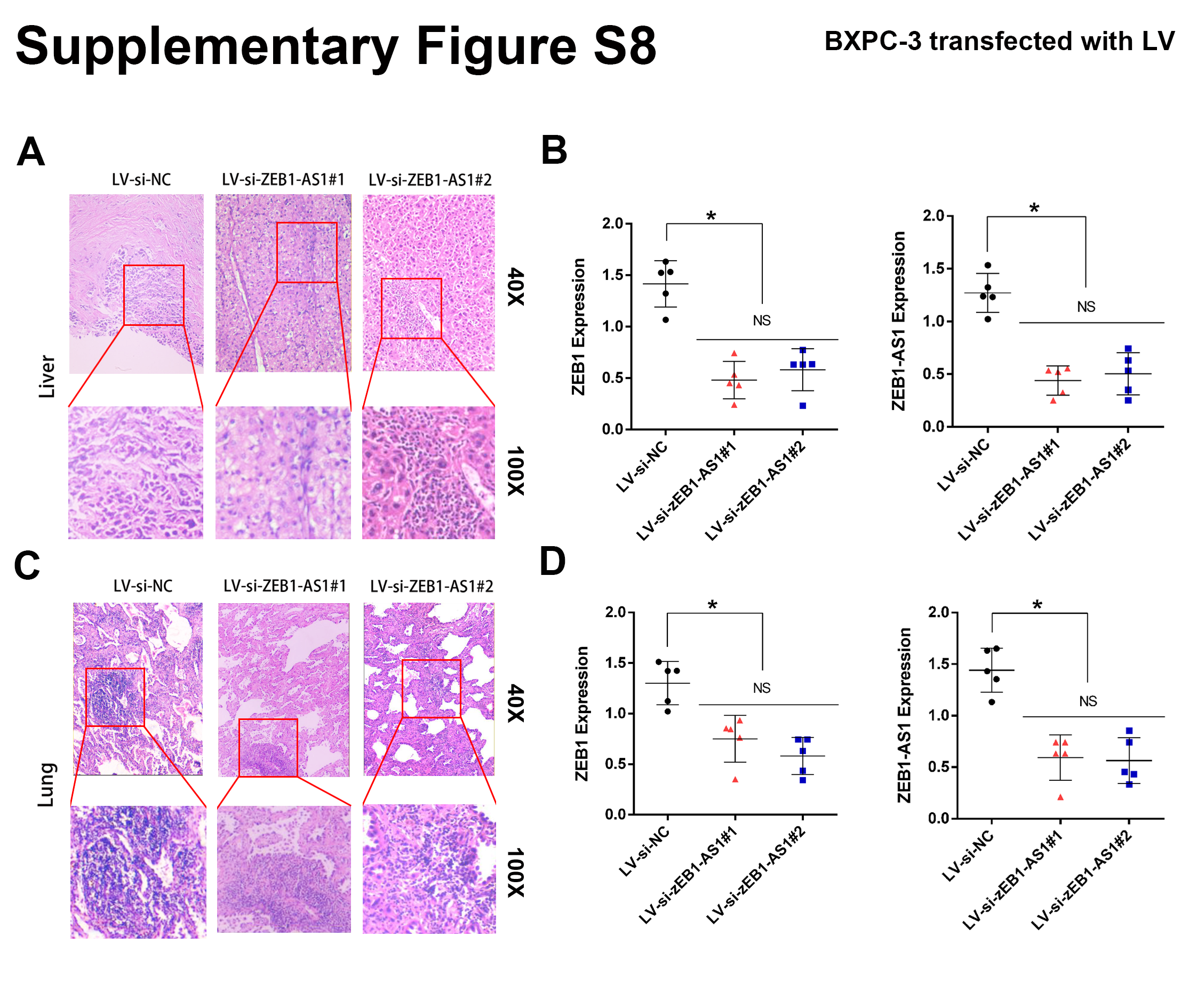

Supplement: Supplementary Figure 8 — ZEB1-AS1 promotes the PC metastasis of PANC-1 cells in mice. H&E images of liver extracted from LV-si-ZEB1-AS1#1, LV-si-ZEB1-AS1#2 or LV-siNC group of PANC-1 cells in mice. Scale bars, 100 μm. (B) The ZEB1 and ZEB1-AS1 expression were measured via qRT-PCR from the liver metastases. (C) Detecting H&E-stained images for lung tissue extracted from LV-si-ZEB1-AS1#1, LV-si-ZEB1-AS1#2 or LV-siNC group. The scale bars displayed 100 μm in the diagrams. (D) RNA expression level of ZEB1 and ZEB1-AS1 were evaluated from the lung metastases. quantitative data were manifested as means ± SD of at least three experiments independently. Values with statistic difference are significant at *P < 0.05 and **P < 0.01 as marked significance. [file Image_8.tif]
