# Supplementary material for: Positive Reciprocal Feedback of lncRNA ZEB1-AS1 and HIF-1α Contributes to Hypoxia-Promoted Tumorigenesis and Metastasis of Pancreatic Cancer
Source: Front Oncol. 2021 Nov 22;11:761979. doi: 10.3389/fonc.2021.761979 (PMC8645903; doi:10.3389/fonc.2021.761979)
Supplement: Supplementary file 9 [file Table_1.doc]

**Supplementary Table S1: The sequences of siRNAs.**

| **SiRNA Targets** | **Sequences** |
| --- | --- |
| HIF-1α-siRNA#1 sense | 5′-CUGAUGACCAGCAACUUGA-3 |
| HIF-1α-siRNA#2 sense | 5′-CAAUCAAGAAGUUGCAUUA-3′ |
| HIF-1α-siRNA#3 sense | 5′-CUGAUGACCAGCAACUUGA-3′ |
| ZEB1-AS1-siRNA#1 sense | 5′-GGAGCCATCTAGTGCATAA-3′ |
| ZEB1-AS1-siRNA#2 sense | 5′-GGACCAACTTTATGGAATA-3′ |
| ZEB1-AS1-siRNA#3 sense | 5′-GCTGAAGTCTGATGATTTA-3′ |
| ZEB1-siRNA#1 sense | 5′-GGCAAGTGTTGGAGAATAA-3′ |
| ZEB1-siRNA#2 sense | 5′-GGACAGCACAGTAAATCTA-3′ |
| ZEB1-siRNA#3 sense | 5′-CGGACGAGAGAGAGAGTTT-3′ |
| HDAC1-siRNA#1 sense | 5′-CTGGCAAAGGCAAGTA-3′ |
| HDAC1-siRNA#2 sense | 5′-CCGCAAGAACTCTTCC-3′ |
| HDAC1-siRNA#3 sense | 5′-CTTTGGAAAGGTGCCC-3′ |
| NC-siRNA sense | 5′-UUCUCCGAACGUGUCACGUUU-3′ |
