# Supplementary material for: Positive Reciprocal Feedback of lncRNA ZEB1-AS1 and HIF-1α Contributes to Hypoxia-Promoted Tumorigenesis and Metastasis of Pancreatic Cancer
Source: Front Oncol. 2021 Nov 22;11:761979. doi: 10.3389/fonc.2021.761979 (PMC8645903; doi:10.3389/fonc.2021.761979)
Supplement: Supplementary file 10 [file Table_2.doc]

**Supplementary Table S2: The sequences of PCR primers.**

| **Primers** | **Sequences** |
| --- | --- |
| ZEB1-AS1 | Forward: 5′-GAACCGGGATGGGAAGTGAC-3′ |
|  | Reverse: 5′-GCAAGCGGAACTTCTAGCCT-3′ |
| HIF-1α | Forward: 5′-GACAAGCCACCTGAGGAGAG-3′ |
|  | Reverse: 5′-GTGGCAACTGATGAGCAAGC-3′ |
| ZEB1 | Forward: 5′-CGCAGTCTGGGTGTAATCGT-3′ |
|  | Reverse: 5′-TTGCAGTTTGGGCATTCATA-3′ |
| HDAC1 | Forward: 5′-ACGACGGGGATGTTGGAAATTA-3′ |
|  | Reverse: 5′-CAGCATTGGCTTTGTGAGGG-3′ |
| β-actin | Forward: 5′-CATGTACGTTGCTATCCAGGC-3′ |
|  | Reverse: 5′-CTCCTTAATGTCACGCACGAT-3′ |
| GAPDH | Forward: 5′-CCCCGGTTTCTATAAATTGAGC-3′ |
|  | Reverse: 5′-CACCTTCCCCATGGTGTCT-3′ |
| U6 | Forward: 5′-CTCGCTTCGGCAGCACA-3′ |
|  | Reverse: 5′-AACGCTTCACGAATTTGCGT-3′ |
| ChIP primers for ZEB1-AS1 | Forward:5′-AAGCAACAGCCGCTCCAC-3′ |
|  | Reverse: 5′-CTCCCTGGACCGTTAGCC-3′ |
| ChIP primers for HIF-1α | Forward:5′-CTGTCCGCTAAGGTCAAT-3′ |
|  | Reverse: 5′-AAGCATCAAACTCTGACAAG-3′ |
